# Adverse childhood experiences, daytime salivary cortisol, and depressive symptoms in early adulthood: a longitudinal genetically informed twin study

**DOI:** 10.1038/s41398-021-01538-w

**Published:** 2021-08-05

**Authors:** Eleonora Iob, Jessie R. Baldwin, Robert Plomin, Andrew Steptoe

**Affiliations:** 1 Research Department of Behavioural Science and Health, Institute of Epidemiology and Healthcare, University College London, London, UK; 2 Division of Psychology and Language Sciences, University College London, London, UK; 3 Social, Genetic and Developmental Psychiatry Centre, Institute of Psychiatry, King’s College London, London, UK

**Keywords:** Depression, Prognostic markers

## Abstract

Dysregulated hypothalamic–pituitary–adrenal (HPA)-axis function might underlie the relationship between adverse childhood experiences (ACEs) and depression. However, limited research has examined the possible mediating role of the HPA-axis among young people using longitudinal data. Moreover, it remains unclear whether genetic influences could contribute to these associations. Participants were 290 children from the Twins Early Development Study. ACEs were assessed from age 3–11 years. We calculated a cumulative risk score and also derived different ACEs clusters using factor analysis and latent class analysis. HPA-axis activity was indexed by daytime salivary cortisol at age 11. Depressive symptoms were ascertained at age 21. Genetic liability to altered cortisol levels and elevated depressive symptoms was measured using a twin-based method. We performed causal mediation analysis with mixed-effects regression models. The results showed that ACEs cumulative exposure (*b* = −0.20, *p* = 0.03), bullying (*b* = −0.61, *p* = 0.01), and emotional abuse (*b* = −0.84, *p* = 0.02) were associated with lower cortisol levels at age 11. Among participants exposed to multiple ACEs, lower cortisol was related to higher depressive symptoms at age 21 (*b* = −0.56, *p* = 0.05). Lower cortisol levels mediated around 10–20% of the total associations of ACEs cumulative exposure, bullying, and dysfunctional parenting/emotional abuse with higher depressive symptoms. Genetic factors contributed to these associations, but the mediation effects of cortisol in the associations of ACEs cumulative exposure (*b* = 0.16 [0.02–0.34]) and bullying (*b* = 0.18 [0.01–0.43]) remained when genetic confounding was accounted for. In conclusion, ACEs were linked to elevated depressive symptoms in early adulthood partly through lower cortisol levels in early adolescence, and these relationships were independent of genetic confounding.

## Introduction

Adverse childhood experiences (ACEs), including both the absence of stimulation needed for typical development and the presence of harmful or threatening stimulation [1], are very common in several countries [2, 3]. In addition, they have been linked with a higher risk of mental disorders, chronic physical health conditions (e.g. cardiovascular disease and cancer) and health-harming behaviours [4]. The first major studies about the effects of ACEs on health and child development considered adversities such as abuse (emotional, physical and sexual), neglect (emotional and physical) and experiences of household dysfunction (e.g. violence between parents and parental separation) [5]. More recently, studies on ACEs have also incorporated additional adversities to represent other important domains of childhood experiences that might be important in predicting long-term health and well-being outcomes [6], such as parent–child relationships, parenting styles and bullying [7]. Stress-related psychopathologies including depression are the health outcomes most strongly related to ACEs. For instance, individuals exposed to multiple ACEs have a threefold increased risk of depression [8, 9]. Given their high prevalence, lifelong health consequences, and associated societal costs, understanding the risk and protective mechanisms underlying the impact of ACEs on mental health is an important research priority that might stimulate more targeted preventive strategies.

The hypothalamic–pituitary–adrenal (HPA)-axis is thought to be one of the main neurobiological mechanisms through which ACEs could lead to the emergence of stress-related psychopathology [10, 11]. Repeated or excessive exposure to psychosocial stressors has been linked to chronic alterations in the function of the HPA-axis, with detrimental physiological consequences for the brain and other body systems [12]. This is particularly the case for children and young people exposed to stressful and traumatic experiences, whose brain structures are still developing at the time of stress exposure [10]. A large number of observational studies have investigated the relationship of ACEs with the HPA-axis, including basal functioning and response to social stress tasks. Overall, this work has provided important evidence for dysregulated levels of cortisol (i.e. the primary end product of the HPA-axis) in children and adults exposed to ACEs [11, 13]. However, there is little agreement on the direction of these associations. Early research using salivary cortisol implied that ACEs were linked to increased stress-induced cortisol secretion and elevated basal cortisol levels [14–16], but a growing body of studies now also suggest reduced HPA-axis activation [17–19]. Similar inconsistencies have also been documented in studies measuring cortisol in hair, which provides an indication of average cortisol exposure over several weeks [20, 21]. Hence, other factors than the measurement of cortisol are likely to be involved.

Different lines of research suggest that abnormal HPA-axis functioning may be implicated in the aetiology of stress-related disorders including depression. Several investigations have demonstrated that depressed individuals tend to exhibit HPA-axis hyperactivation [22]. In addition, elevated cortisol levels have been shown to predict the risk of future depression onset in healthy individuals [23]. Interestingly, different patterns of HPA-axis activity have been found in other stress-related disorders. Notably, patients with post-traumatic stress disorder (PTSD) and those with PTSD and comorbid major depressive disorder (MDD) have been shown to have lower salivary cortisol levels than controls [24, 25]. Consequently, depressed individuals exposed to traumatic events could exhibit different patterns of HPA-axis activity compared with those without trauma.

Despite the large number of studies investigating the role of the HPA-axis in the link between ACEs and stress-related psychopathologies, several uncertainties currently remain. First, the timing of HPA-axis development could play an important role in the relationship between stress exposure and cortisol levels. While early childhood seems to be characterised by a hyporesponsive stress response [10], the HPA-axis has been shown to enter a phase of hypersensitivity to the environment with the beginning of puberty during late childhood or adolescence [13]. Importantly, exposure to ACEs could alter these typical patterns of HPA-axis activity found across different early life periods. For instance, a number of studies have found that exposure to ACEs, such as maltreatment and family dysfunction, was associated with elevated HPA-axis responses during early childhood, but the same types of adversities were related to reduced HPA-axis activity during late childhood and adolescence [26, 27]. Second, the vast majority of studies have used cross-sectional designs and did not formally test the potential role of the HPA-axis as a mediating mechanism through which ACEs might increase the risk of later stress-related psychopathology. Third, it remains unclear to what extent genetic influences might contribute to these relationships. HPA-axis function and depression are both influenced by genetic factors, which could increase the individual’s vulnerability to ACEs. Twin-based heritability estimates have been shown to range between 30 and 40% for depression [28] and between 40 and 60% for salivary cortisol [29, 30]. Genes contributing to stress-related disorders could also affect the risk of exposure to ACEs through passive, evocative, or active gene-environment correlations [31]. Yet, few studies have tested whether the associations of ACEs and cortisol with depression are attenuated or confounded by genetic liability. Lastly, different types or patterns of ACEs might be related to distinct physiological and psychological effects on child development [1]. It has been proposed that the HPA-axis and immune systems might respond differently to different types of early life stressors [13, 32, 33]. For example, some preliminary evidence suggests that experiences related to threat (e.g. abuse and interparental conflict) but not deprivation (e.g. poverty) are associated with altered HPA-axis activity [34, 35]. However, studies to date have typically investigated ACEs either by calculating cumulative risk scores (i.e. total number of adversities experienced by the participant), or by testing the effects of few selected adversities in isolation. Importantly, both methods ignore the potential clustering of ACEs [36]. Hence, there is a need to explore the role of distinct ACEs patterns in order to understand whether specific types or combinations of ACEs might be more strongly related to psychopathology than others.

In order to address these knowledge gaps, we compared the longitudinal associations of cumulative risk scores and clusters of ACEs with salivary cortisol levels during adolescence and with the emergence of depressive symptoms in early adulthood, while controlling for genetic liability to cortisol and depressive symptoms using a twin-based method. In addition, we used mediation analysis to assess whether cortisol dysregulations might underlie the association between ACEs and depressive symptoms.

## Materials and methods

### Sample

The data came from a subsample of participants from the Twins Early Development Study (TEDS). TEDS is a longitudinal cohort study of twins born in England and Wales between 1994 and 1996, whose aim is to disentangle genetic and environmental influences on cognitive and behavioural development [37]. Data collection took place at multiple time points from infancy up to early adulthood (i.e. 21 years). The subsample used for this investigation was part of a follow-up study focusing on environmental determinants of childhood adiposity and eating behaviours, as described elsewhere [30]. Briefly, 173 families with same-sex twins were visited at home by trained researchers when the twins were aged 11 years, on average. Cortisol samples were collected from 150 twin pairs, including 80 monozygotic (MZ) and 70 dizygotic (DZ) pairs (*N* = 300). This sample has adequate power to study genetic and early life environmental influences on cortisol [30, 38]. There were no significant differences in the baseline characteristics of twins who took part in the cortisol study compared with the full TEDS sample (Supplementary Table 1 for different tests and *p*-values). Ethical approval was obtained from the research ethics committees of King’s College London and University College London, and all families provided written informed consent.

### Measures

#### Adverse childhood experiences

Exposure to several ACEs between age 3 and 11 years was assessed repeatedly using both prospectively and retrospectively collected information, reported by the parents and the twins. These included negative parental practices, negative parental feelings, maternal depression, divorce of parents, separation from parents, bullying, emotional abuse and physical abuse. The selection of these ACEs exposures was informed by the ACEs definition proposed by McLaughlin and Sheridan [1], and by recent studies in this field which have considered a wider range of adversities compared with the first major ACEs investigations to provide a more comprehensive assessment of this construct. A detailed description of the questionnaires used to measure ACEs and scoring methods can be found in the Appendix (Supplementary Methods and Supplementary Tables 2–6). Supplementary Table 7 (Appendix) also provides an overview of the available time points, sources (i.e. parents or twins), and methods (i.e. prospective or retrospective) of data collection for each ACE. For this analysis, we derived a binary indicator combining data from all available assessments for each type of ACE. For the numerical ACEs measures (i.e. negative parental practices, negative parental feelings, bullying, emotional abuse and physical abuse), we calculated the mean score across different time points and then derived a binary variable indicating ‘high exposure’ using the highest tertile. For the binary ACEs measures (i.e. divorce, separation and maternal depression), we derived a binary variable representing any exposure to the relevant ACE throughout childhood. Using these binary indicators, we then compared three different ways of operationalising ACEs, including (1) cumulative ACEs scores, (2) clusters of ACEs derived through factor analysis (FA) and (3) clusters of ACEs derived through latent class analysis (LCA) (see ‘Statistical analyses’ section).

#### Salivary cortisol

Participants were visited at home by trained researchers and were invited to play a computer game. Saliva samples were collected before playing the computer game (pre-task), immediately after the game (post-task 1) and 10 min later (post-task 2). All visits took place in the afternoon, and none of the twins had eaten within 3 h of the cortisol assessment. The saliva samples were collected using Salivettes, with each cotton roll held in the mouth for two minutes. These were then stored at −20 °C until the completion of the study. Cortisol levels were measured using a high sensitivity chemiluminescence assay (IBL-Hamburg, Hamburg, Germany) at the Technical University Dresden (Germany). Inter- and intra-assay variance coefficients were <8%. Missing values resulted in 290 out of 300 twins having data analysed. For this study, we calculated two cortisol measures: (1) Average daytime cortisol, computed as the mean cortisol value across pre-task, post-task 1 and post-task 2; (2) cortisol reactivity, computed as the change between pre-task and post-task 1. We focused on these cortisol measures to represent two key components that have been shown to underlie salivary cortisol levels, namely ‘total cortisol production’ and ‘change in cortisol levels’ [39]. The distribution of the cortisol measures in the sample was approximately normal.

#### Depressive symptoms

Depressive symptoms were ascertained using the short mood and feeling questionnaire (SMFQ) [40] when the twins were aged 21 years (see Supplementary Table 8). Each item was scored on a 3-point scale ranging from “Not true” to “Very true”. The total SMFQ score was calculated by adding together all item scores. This scale demonstrates good internal validity in the TEDS sample (*α* = 0.86) [41].

#### Latent genetic risk scores

Genetic liability for cortisol and depressive symptoms was estimated through a latent genetic risk method, which has been widely used to measure additive genetic influences on a trait among twins [42–44]. To construct the two genetic risk scores, we defined low cortisol (given its negative correlations with both ACEs and depressive symptoms) as the lowest tertile of the distribution of the average cortisol measure, and high depressive symptoms as the top tertile of the distribution of the total SMFQ score. We then considered that MZ twins share 100% of their genes, whereas DZ twins typically share 50% of their genetic make-up. Therefore, a child’s genetic liability to cortisol and depressive symptoms was coded as follow: Cortisol—‘lowest risk’ if they had an MZ co-twin without low cortisol, ‘low risk’ if they had a DZ co-twin without low cortisol, ‘high risk’ if they had a DZ co-twin with low cortisol, and ‘highest risk’ if they had an MZ co-twin with low cortisol; Depressive symptoms—‘lowest risk’ if they had an MZ co-twin without high depressive symptoms, ‘low risk’ if they had a DZ co-twin without high depressive symptoms, ‘high risk’ if they had a DZ co-twin with high depressive symptoms, and ‘highest risk’ if they had an MZ co-twin with high depressive symptoms.

#### Covariates

All analyses were adjusted for sex (male or female), age in years, and family socioeconomic status (SES). Family SES was indexed by the mother’s educational level. Higher SES was defined as mothers with A-level equivalent or university degree qualifications.

### Statistical analyses

In order to account for the cumulative effects and patterning of ACEs, three different approaches were used to operationalise ACEs. First, a cumulative ACEs score was created by adding together the total number of ACEs experienced by each participant. This was then categorised into 0 ACEs, 1 ACE, 2 ACEs and 3+ ACEs. Second, FA was used to derive distinct variable-centred ACEs clusters, namely groups of ACEs that tend to co-occur across participants. These were identified by randomly splitting the sample into two halves (i.e. training and test data), and then performing explorative FA (EFA) with the training data using the R package *psych* [45] and confirmatory FA (CFA) with the test data using the R package *lavaan* [46] *and semTools* [47]. This approach helped us to reduce the risk of overfitting. In the analysis, each FA-derived cluster was indexed by a binary variable indicating the presence or absence of any exposure to the relevant ACEs throughout childhood. Third, LCA was performed using the R package *poLCA* [48] in order to derive person-centred ACEs clusters, namely groups of participants co-reporting similar ACEs. Models for 2–6 classes were compared in both the training and test datasets to check whether the same best-fitting class solution emerged in both samples. Following the identification of the optimal class solution, the study participants were assigned to their most likely class thereby creating a categorical variable for use in regression analysis. The application of these two data-driven clustering techniques allowed us to assess the role of different patterns of ACEs, and to understand whether the results differ according to the specific method used to operationalise ACEs. Furthermore, the grouping of ACEs into different clusters typically results in a higher prevalence of ACEs within each group, compared with the prevalence of single ACEs which was generally low in our sample (see ‘Results’ section).

**Fig. 1:**
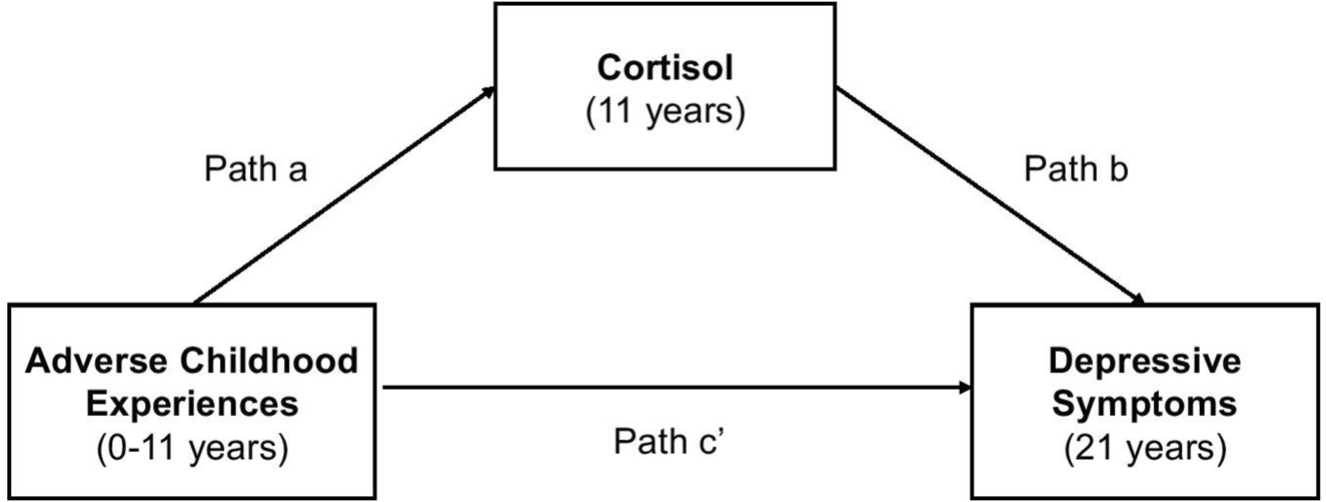
Causal mediation mechanism diagram. *Note.* Illustration of the study design and the causal mediation mechanism.

We applied Cholesky decomposition to determine additive genetic (A), shared environment (C) and non-shared environment (E) influences [49] on cortisol and depressive symptoms, using the *umx* R package for twin and path-based structural equation modelling [50]. We then tested the associations between ACEs (cumulative scores and clusters), cortisol and depressive symptoms using mixed-effects linear regression models with the *lme4* R package [51], and we controlled for genetic influences using the latent genetic scores. The use of mixed-effects models enabled us to account for the paired structure of the data by including a random intercept for each twin pair in the regression models. Post hoc tests did not reveal significant violations of the assumptions of mixed-effects linear models. Further, we performed model-based causal mediation analysis using the *Mediation* R package [52] to examine whether the exposure variable (ACEs) was indirectly associated with the outcome (depressive symptoms) through the mediator (cortisol) (Fig. 1). In our study, the mediator (cortisol) was modelled using two linear mixed-effects models: Model 1—including the exposure (ACEs) and pre-exposure covariates (sex, age and family SES); Model 2—Model 1 + latent genetic risk scores (*a* path, Fig. 1). The outcome (depressive symptoms) was modelled using four linear mixed-effects models: Model 1—including the exposure or mediator and the pre-exposure covariates (sex, age and family SES); Model 2—Model 1 + latent genetic risk scores; Model 3—including both the exposure and the mediator and all pre-exposure covariates (sex, age, family SES and latent genetic risk scores); Model 4—Model 3 + an interaction term between the exposure and mediator (*b* and *c’* paths, Fig. 1). The average causal mediation effects of cortisol (ACMEs) were estimated both without (Model 1) and with (Model 2) adjustment for latent genetic risk scores. In addition, we accounted for exposure-mediator interactions to examine whether the ACMEs may take different values depending on the exposure status (i.e. no exposure vs exposure to ACEs) [52]. Separate mediation models were fitted for the ACEs cumulative score and each ACEs dimension. All models were estimated using bias-corrected bootstrapping with 1000 replications.

Missing data on the outcome and exposure variables were estimated using multiple imputation by chained equations (MICE) with the *mice* R package [53]. The proportions of missing data for all imputed variables are shown in Supplementary Table 9. Further details on FA, LCA, causal mediation, and MICE analyses are provided in the Appendix (Supplementary Methods).

## Results

### Descriptive statistics

The sample characteristics in the observed and imputed data are described in the Appendix (Supplementary Results) and reported in Supplementary Table 9 (Appendix). The prevalence of the individual ACEs items is reported in Supplementary Table 10. The participants’ characteristics in the observed and imputed data were similar, indicating that the MICE procedure achieved its aims.

### FA-derived and LCA-derived ACEs clusters

**Fig. 2:**
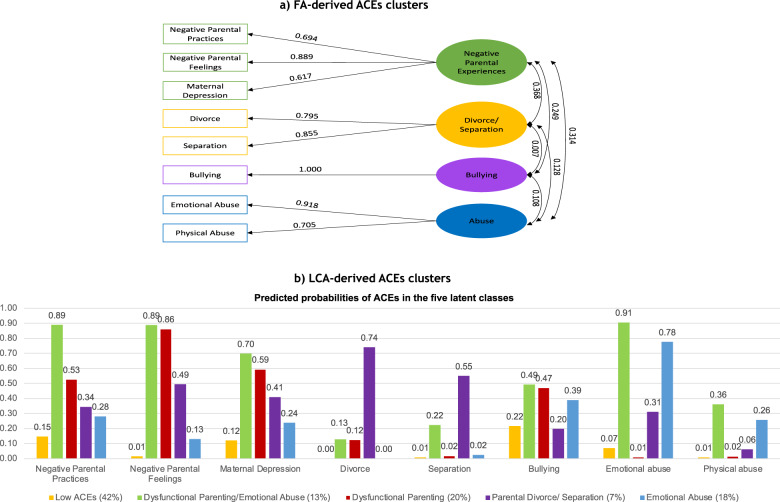
FA-derived and LCA-derived ACEs clusters. *Note*. **a** Standardised factor loadings of the ACEs items in the CFA model. **b** Predicted probabilities of the ACEs items in the five-class LCA model. CFA confirmatory factor analysis, LCA latent class analysis, ACEs adverse childhood experiences.

The statistical results of the EFA of ACEs in the training dataset are shown in Supplementary Table 11 (Appendix). The final CFA model fitted on the test dataset is illustrated in Fig. 2a. The model fit the test dataset well, SRMSR = 0.063, RMSEA = 0.047, CFI = 0.974 and TLI = 0.949. Standardised factor loadings were high for all ACEs indicators (range: 0.617–1.000). All factors had good discriminant validity since their correlations were lower than 0.85 [54]. This analysis revealed four distinct dimensions of ACEs, namely: *dysfunctional parenting* (i.e. negative parental practices/feelings and maternal depression), *parental divorce/separation*, *bullying* and *abuse* (physical or emotional). Bullying was included as a standalone dimension in the final CFA model since it did not correlate well with any of the latent factors (i.e. factor loadings on all latent factors were less than 0.3) [55]. To further validate the final CFA model, we also tested its measurement invariance across the training and test datasets. The results provided evidence for both weak and strong measurement invariance (see Supplementary Table 12).

Regarding LCA, the five-class solution was found to be the best fitting solution in both the training and test datasets. The model fit of the 2–6 class models in the training and test datasets is shown in Supplementary Table 13 (Appendix). The predicted probabilities of each ACE in each of the five classes are shown in Fig. 2b. The *Low ACEs* class was the largest group (42%) and comprised of participants with a low probability (<0.5) of any ACEs. The *dysfunctional parenting/emotional abuse* class (13%) included participants with a high probability (>0.5) of reporting negative parental practices, negative parental feelings, maternal depression and emotional abuse. The *dysfunctional parenting* class (20%) was comprised of participants with a high probability of reporting negative parental practices, negative parental feelings and maternal depression. The *parental divorce/separation* class was the smallest group (7%) and included participants with a high probability of parental divorce and separation. Lastly, the *emotional abuse* class (18%) represented participants with a high probability of reporting emotional abuse, but low probabilities of other ACEs. Bullying and physical abuse did not cluster well with other ACEs in this sample as the probability of endorsing these items was low in all classes.

### Genetic influences on cortisol and depressive symptoms

The intraclass correlation coefficient (ICC) of average cortisol (i.e. ratio of between-twin pair variance over total variance) was 0.445, suggesting that around 45% of its variance was accounted for by within-twin pair correlations. Cholesky decomposition further indicated that 44% of the observed variation in cortisol levels was explained by additive genetic effects, 15% was explained by the twins’ shared environment, and 40% by non-shared environmental influences (Supplementary Fig. 1, Appendix). These results are consistent with earlier heritability estimates of salivary cortisol found in this and other twin samples [29, 30]. The ICC of depressive symptoms was 0.519, indicating that around 50% of its variance was explained by within-twin pair correlations. Cholesky decomposition showed that 37% of the observed variation in depressive symptoms was explained by additive genetic effects, 13% was explained by shared environmental influences, and 50% by non-shared environmental effects (Supplementary Fig. 1, Appendix), as suggested by previous twin-based heritability studies of depression [28, 56].

### Associations of ACEs with average daytime cortisol levels in adolescence (path a)

**Fig. 3:**
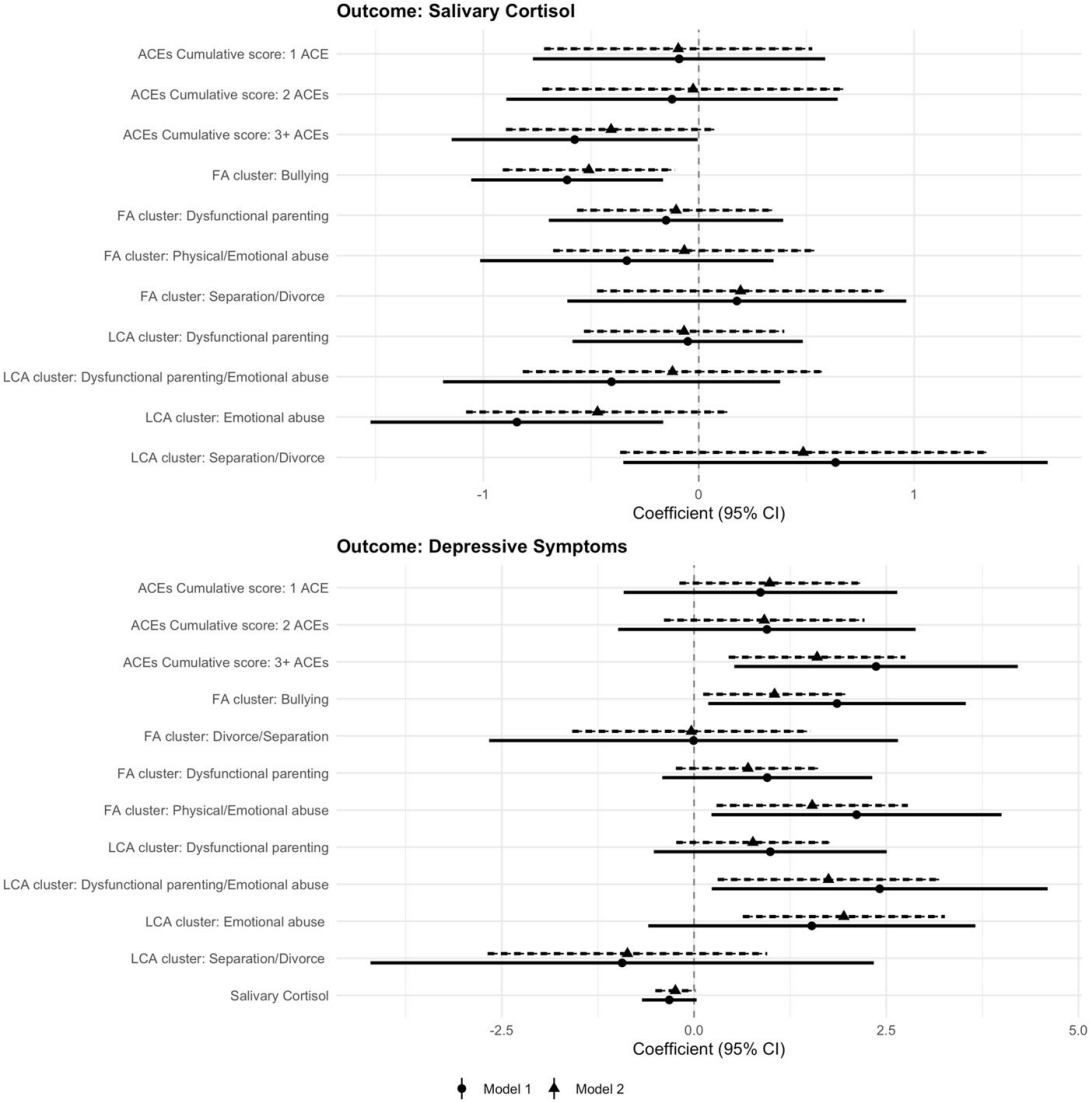
Associations between ACEs, average daytime cortisol levels (age 11) and depressive symptoms (age 21). *Note.* *N* = 290. Pooled estimates from mixed-effects linear regression analysis across 20 imputed datasets. Model 1 = adjusted for sex, age and family SES; Model 2 = Model 1 + latent genetic risk scores for cortisol/depressive symptoms. Reference groups: ACEs cumulative score = ‘0 ACEs’; LCA-derived clusters: ‘Low ACEs’. ACEs adverse childhood experiences, SES socioeconomic status, CI confidence interval.

The associations of ACEs with average cortisol levels at age 11 are illustrated in Fig. 3 and reported in Supplementary Table 14 (Appendix). In the model adjusted for sex, age and family SES (Model 1), children exposed to 3+ ACEs had significantly lower cortisol levels than those who did not have any ACE (*b* = −0.576, *p* = 0.049), although the effect size of this association was small (*β* = −0.066). Among the FA-derived ACEs clusters, *bullying* victimisation exhibited the largest relationship with cortisol (*b* = −0.611, *p* = 0.008, *β* = −0.061), whereas other ACEs clusters (i.e. abuse, parental divorce/separation and dysfunctional parenting) were not independently associated with cortisol. With regard to the LCA-derived ACEs clusters, the *emotional abuse* group had significantly lower cortisol levels than the *low ACEs* group (*b* = −0.844, *p* = 0.016, *β* = −0.085), but no significant differences were found between other ACEs clusters. The relationships of ACEs with average cortisol levels were attenuated after adjustment for genetic liability for cortisol, but a significant yet small association with bullying remained (Model 2: *b*_ACE:3+_ = −0.407, *p* = 0.100, *β* = −0.045; *b*_Bullying_ = −0.510, *p* = 0.013, *β* = −0.051; *b*_EmotionalAbuse_ = −0.470, *p* = 0.133, *β* = −0.047).

### Associations of average daytime cortisol levels and ACEs with depressive symptoms in early adulthood (paths b and c’)

**Fig. 4:**
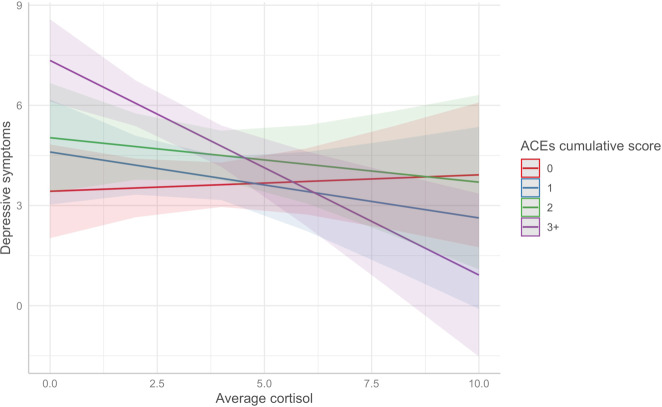
Interaction effect between ACEs cumulative exposure and average daytime cortisol levels on depressive symptoms. *Note*. Model 4—adjusted for sex, age, family SES and latent genetic risk scores. ACEs adverse childhood experiences, SES socioeconomic status.

The associations of average cortisol levels and ACEs with depressive symptoms at age 21 are illustrated in Fig. 3 and reported in Supplementary Table 15 (Appendix). Lower cortisol levels were related to higher depressive symptoms, but this association was only borderline significant (*b* = −0.322, *p* = 0.072, *β* = −0.20). Children exposed to 3+ ACEs had higher depressive symptoms at age 21, independently of genetic liability and other covariates (Model 2: *b*_ACE:3+_ = 1.601, *p* = 0.007, *β* = 0.132). Among the FA-derived ACEs clusters, *abuse and bullying* exhibited the largest associations with higher depressive symptoms, which remained also when accounting for genetic liability (Model 2: *b*_Abuse_ = 1.537, *p* = 0.017, *β* = 0.096; *b*_Bullying_ = 1.047, *p* = 0.030; *β* = 0.065). In addition, children in the LCA-derived groups representing *dysfunctional parenting/emotional abuse* and *emotional abuse* had significantly higher depressive symptoms than those in the *low ACEs* group, independently of genetic liability and other covariates (Model 2: *b*_Dysfunctional Parenting/Emotional Abuse_ = 1.749, *p* = 0.019, *β* = 0.109; *b*_EmotionalAbuse_ = 1.949, *p* = 0.004, *β* = 0.122). Further, we tested interaction effects between ACEs and average cortisol on depressive symptoms (Model 4, Supplementary Table 16). We found a small negative interaction effect between exposure to 3+ ACEs and cortisol (*b* = −0.564, *p* = 0.050, *β* = −0.035), independently of genetic liability and other covariates. As illustrated in Fig. 4, lower cortisol levels were associated with higher depressive symptoms among the group of children exposed to 3+ ACEs, but this association was much smaller among children exposed to smaller numbers of ACEs.

### Mediation effects of average daytime cortisol levels in the relationship between ACEs and depressive symptoms

**Fig. 5:**
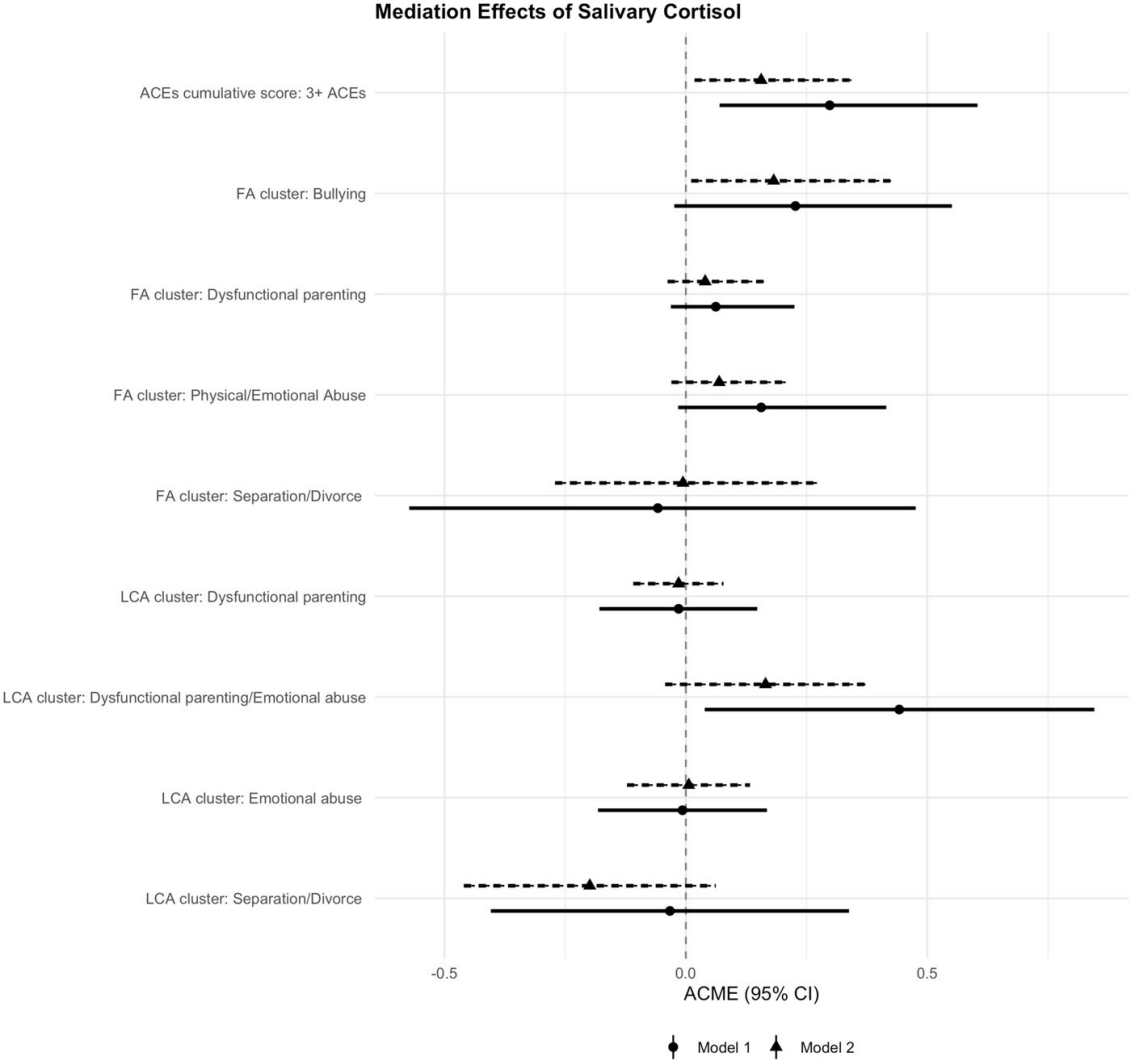
Mediation effects of average daytime cortisol levels in the associations of ACEs with depressive symptoms (whole sample). *Note.* Model 1 = adjusted for sex, age and family SES, Model 2—Model 1 + latent genetic risk scores for cortisol and depressive symptoms. ACME average causal mediation effect, CI confidence interval, ACEs adverse childhood experiences, SES socioeconomic status. A significant mediation effect of cortisol in the association between ACEs and depressive symptoms is present when the CI of the ACME does not cross zero.

The ACMEs of average cortisol levels are reported in Supplementary Table 17 (Appendix) and illustrated in Fig. 5. The ACMEs were calculated in the whole sample, as well as in the groups of participants exposed and unexposed to ACEs separately to account for possible exposure–mediator interactions. In the whole sample, cumulative exposure to 3+ ACEs increased the risk of higher depressive symptoms partly through lower cortisol levels, independently of genetic liability and other covariates (Model 2: ACME = 0.156, 95% CI: 0.018–0.343). Hence, cortisol explained around 11% of the total association of exposure to 3+ ACEs with depressive symptoms (Supplementary Table 17). When accounting for the interaction between ACEs and cortisol, the results showed that the ACME of cortisol took different values according to the exposure status. Specifically, this effect was almost twice as large in the group of participants exposed to 3+ ACEs (proportion-mediated effect = 20%), while it was nonsignificant in the unexposed group (Supplementary Table 17). This suggests that cortisol was more strongly related to depressive symptoms in children exposed to ACEs, as indicated by the interaction effects presented above (Fig. 4). We also found a significant ACME of cortisol in the association of *bullying* with depressive symptoms in the whole sample independently of genetic liability and other covariates (Model 2: ACME = 0.182, 95% CI: 0.011–0.425), suggesting that cortisol mediated 20% of the total association of bullying with depressive symptoms (Supplementary Table 17). In addition, cortisol mediated 18% of the total association between the LCA-derived cluster *dysfunctional parenting/emotional abuse* and depressive symptoms (Model 1: ACME = 0.442, 95% CI: 0.039–0.846), although this effect was no longer significant after adjustment for genetic liability. The ACMEs of cortisol for other ACEs clusters were considerably smaller and nonsignificant in all models.

### Associations between ACEs, cortisol reactivity and depressive symptoms

The ACEs cumulative score and the FA-derived ACEs clusters were not associated with cortisol reactivity (Supplementary Table 18). The LCA-derived cluster *dysfunctional parenting/emotional abuse* was associated with lower cortisol reactivity (Model 2: *b* = −0.654, *p* = 0.036, *β* = −0.061), while the LCA-derived cluster representing *dysfunctional parenting* was associated with higher cortisol reactivity (*b* = 0.468, *p* = 0.021, *β* = 0.043), independently of genetic liability and other covariates. Despite these associations, cortisol reactivity did not predict depressive symptoms (Supplementary Table 19), and its interaction effects with all ACEs variables were not related to differences in depressive symptoms. Further, all mediation effects of cortisol reactivity in the associations between ACEs and depressive symptoms were small and nonsignificant (Supplementary Table 20).

### Sensitivity analyses

We conducted several sensitivity analyses in order to assess the robustness of the results of the main imputed data analysis. First, we tested the associations of the FA-derived ACEs clusters with cortisol levels and depressive symptoms using a latent multilevel structural equation model (SEM), rather than regression analysis, in order to account for the intercorrelations among the ACEs items. The associations with cortisol mirrored those found in the regression analysis as only *bullying* was associated with lower cortisol levels. As for the regression analysis, *abuse* and *bullying* were associated with higher depressive symptoms in the SEM model. In addition, there was a positive association between *dysfunctional parenting* and depressive symptoms, which was not found in the regression analysis (Supplementary Table 21). Nevertheless, this is consistent with the results of the main analysis using the LCA-derived clusters, which showed that the *dysfunctional parenting/emotional abuse* cluster was related to higher depressive symptoms. Second, for transparency, we also tested the associations of the individual ACEs items with cortisol and depressive symptoms. As expected, only bullying was significantly associated with lower cortisol levels, and there was a borderline significant association with negative parental practices (Supplementary Table 22). Further, bullying, emotional abuse, and physical abuse were all associated with higher depressive symptoms. In addition, negative parental practices and maternal depression exhibited borderline significant associations with depressive symptoms (Supplementary Table 22). Third, the mediation effects of average cortisol in the associations of the ACEs cumulative score and bullying victimisation with depressive symptoms at age 21 were not affected by earlier depressive symptoms (Supplementary Table 23). This provides further evidence for the plausible causal effect of cortisol on depressive symptoms. Fourth, we retested the associations between ACEs, cortisol levels, and depressive symptoms in the sample of participants with complete data on all variables (*N* = 163). The patterns of associations were broadly similar to those found in the imputed data analysis, but the standard errors and *p*-values were generally larger owing to the reduced sample size of the complete dataset (Supplementary Tables 24 and 25). Lastly, we calculated *E*-values for all statistically significant associations found in the most restrictive models of the main imputed data analysis [57]. The *E*-values were not particularly large (see Supplementary Tables 14, 15 and 18), suggesting that modest unmeasured confounding effects could explain away these associations. However, the analysis did adjust for the most likely confounders of the relationships between ACEs, cortisol, and depressive symptoms, including demographic, socioeconomic and genetic characteristics.

## Discussion

### Summary of main findings

Our analysis revealed several key findings. Children exposed to three or more ACEs had lower daytime cortisol levels at age 11 and elevated depressive symptoms at age 21. Among different types and clusters of ACEs, bullying and emotional abuse had the largest associations with cortisol, and the clusters representing emotional/physical abuse, dysfunctional parenting/emotional abuse and bullying showed the largest associations with depressive symptoms. Lower cortisol was also associated with higher depressive symptoms at age 21 among participants exposed to multiple ACEs. Further, the mediation analysis indicated that cortisol mediated around 10–20% of the total associations of ACEs cumulative exposure, bullying, and dysfunctional parenting/emotional abuse with depressive symptoms. The relationships among ACEs, cortisol and depressive symptoms were generally attenuated when controlling for genetic liability, but both ACEs and cortisol remained as risk factors for later depressive symptoms.

### Interpretation of the findings in relation to previous evidence

Our analysis suggests that ACEs are linked with lower HPA-axis diurnal activity in early adolescence. Previous studies have demonstrated that adolescents exposed to traumatic events had diminished cortisol levels, but this association was not found among younger children [26, 27]. These findings and the results presented here provide some preliminary evidence for a negative association between ACEs and cortisol in early adolescence, a period in which cortisol levels tend to increase with the onset of puberty. We also found that lower cortisol levels during adolescence were associated with higher depressive symptoms in early adulthood, particularly among individuals exposed to ACEs. This finding contradicts previous research suggesting that depressed individuals and people with elevated depressive symptoms tend to exhibit HPA-axis hyperactivation [22, 58]. However, it is partly consistent with earlier analyses showing that PTSD patients with comorbid MDD have lower cortisol levels [25]. Moreover, the majority of these studies focused on middle-aged and older adults, but the relationship of cortisol with depression could be different among children and young people.

Few studies have formally tested whether cortisol dysregulations might underlie the effect of ACEs on the later risk of depression. Our study extends this work by showing that lower cortisol levels explained between 10 and 20% of the total association of ACEs with depressive symptoms in young adulthood. This supports the idea that the HPA-axis might be an important neurobiological mechanism through which ACEs become biologically embedded and increase the individual’s vulnerability to stress-related psychopathology [11].

Our study also expands previous knowledge by assessing the contribution of genetic factors. The results presented here show that genetic liability attenuated these relationships, but did not fully explain them. Hence, both ACEs and genetic liability are likely to be important risk factors for abnormal HPA-axis functioning and depression. These findings are partly consistent with the diathesis-stress model and the differential susceptibility theory, which suggest that certain individuals might be more vulnerable or sensitive to environmental influences as a function of their biological and psychological make-up [11, 59]. It is worth noting that the latent genetic risk scores used in the analysis enabled us to account for potential confounding effects due to gene–environment correlations and interactions between genetic and shared environment (i.e. environments shared between twins) influences. However, we were not able to account for variance attributable to gene by non-shared environment (i.e. environments that differ between twins) interactions.

With regard to the effects of distinct types and clusters of ACEs, experiences involving threat (i.e. bullying and physical and emotional abuse) were associated with both cortisol dysregulations in adolescence and depressive symptoms in young adulthood, while less threatening experiences (parental divorce/separation) were not. Interestingly, experiences of dysfunctional parenting showed weak associations with depressive symptoms, but the combined experience of dysfunctional parenting and emotional abuse was associated with an increased risk of depression. These findings are in line with the results of recent meta-analyses suggesting that emotional abuse is one of the strongest predictors of depression [60–63]. In addition, they support previous research showing that experiences related to threat but not deprivation were associated with altered HPA-axis activity [34, 35]. Nevertheless, these differential associations among different types/clusters of ACEs could be explained by the fact that physical and emotional abuse were retrospectively self-reported at the same time as depression assessment, whereas dysfunctional parenting, divorce/separation and bullying were assessed prospectively. Previous research has found low agreement between prospective and retrospective measures of ACEs [64], with retrospective measures showing stronger associations with psychopathology than prospective measures [65, 66]. Therefore, the association between physical and emotional abuse with depression might reflect retrospective recall bias, whereby individuals with depression are more likely to report abusive experiences due to negative bias in autobiographical memory. To understand whether these findings reflect the effects of different ACEs or the assessment method, future research should test whether prospective and retrospective measures of the same ACEs are differentially associated with health outcomes and mechanisms.

Lastly, the results presented here show that the ACEs cluster representing dysfunctional parenting/emotional abuse was associated with lower cortisol reactivity, whereas the cluster representing only experiences of dysfunctional parenting was associated with higher cortisol reactivity. This result suggests that different clusters of ACEs might be linked to different patterns of HPA-axis activity. However, our measure of cortisol reactivity was unrelated to depressive symptoms, and we found no evidence for its mediating role in the associations between ACEs and depressive symptoms. This could be due to the fact that the game task used in the study did not trigger substantial changes in cortisol responses, although it did elicit marked increases in subjective ratings of excitement [30]. Larger studies are needed to investigate the associations between ACEs clusters, cortisol reactivity and depressive symptoms.

### Strengths, limitations and suggestions for future research

Our analysis has several strengths, including its longitudinal design, adjustment for genetic confounding, measurement of ACEs using both cumulative risk and clustering approaches (i.e. FA and LCA), and assessment of mediation effects using causal mediation analysis. However, our findings should be considered in light of a number of limitations. First, the small sample size of the study might have reduced the power of the moderation and mediation analyses to detect statistically significant differences. Second, the scale measuring negative parental practices has low internal validity in the TEDS cohort, and the dichotomisation of the ACE constructs might have resulted in the loss of some information. Third, although we used multiple measures of cortisol, all assessments took place over a single day. Fourth, as discussed above, our measures of physical and emotional abuse were measured through a retrospective questionnaire and could therefore be disproportionally affected by measurement error. Lastly, the genetic scores were constructed using a latent twin-based method, rather than actual differences in candidate genes or polygenic risk scores. Possible directions for future research include to replicate our results using larger samples of twins and unrelated individuals; test the associations of parenting practices with cortisol and depressive symptoms using a different parenting scale with higher internal validity; assess the role of ACEs, cortisol and genetic factors in other internalising problems, such as anxiety; employ different methods to measure cortisol (e.g. hair cortisol) and genetic vulnerability (e.g. polygenic scores); account for both gene by shared and gene by non-shared environment interactions among twins, and test whether distinct adversity clusters might interact with one another. Another important research priority is to elucidate mechanisms of resilience that could help to prevent stress-related psychopathology in children affected by ACEs, such as stress-adapted skills that children might develop when growing up in unpredictable, harsh environments (e.g. greater attunement to other people and social relationships and enhanced flexibility in switching between tasks) [67].

## Conclusion

To conclude, our study shows that children exposed to multiple ACEs, and particular experiences of abuse and bullying, were more likely to experience elevated depressive symptoms in early adulthood, and this relationship was partly explained by lower cortisol levels in early adolescence. Genetic influences might also contribute to these associations, but ACEs and cortisol are likely to be independent risk factors for depressive symptoms. Intervention studies are needed to test whether early interventions to reduce stress in children affected by ACEs, such as psychotherapy, promotion of physical activity, or pharmacotherapy, could have beneficial effects on the function of the HPA-axis and help to prevent the emergence of stress-related psychopathologies later in life.

## Supplementary information

Appendix file

## Data Availability

The code of the statistical analyses is available from the corresponding author (EI) upon request.

## References

[1] McLaughlin KA, Sheridan MA (2016). Beyond cumulative risk: a dimensional approach to childhood adversity. Curr Dir Psychol Sci.

[2] Merrick MT, Ford DC, Ports KA, Guinn AS (2018). Prevalence of adverse childhood experiences from the 2011-4 behavioral risk factor surveillance system in 23 states. JAMA Pediatr.

[3] Degli Esposti M, Humphreys DK, Jenkins BM, Gasparrini A, Pooley S, Eisner M (2019). Long-term trends in child maltreatment in England and Wales, 1858–2016: an observational, time-series analysis. Lancet Public Health.

[4] Bellis MA, Hughes K, Ford K, Ramos Rodriguez G, Sethi D, Passmore J (2019). Articles Life course health consequences and associated annual costs of adverse childhood experiences across Europe and North America: a systematic review and meta-analysis. Lancet Public Health.

[5] Dong M, Anda RF, Felitti VJ, Dube SR, Williamson DF, Thompson TJ (2004). The interrelatedness of multiple forms of childhood abuse, neglect, and household dysfunction. Child Abus Negl.

[6] Finkelhor D, Shattuck A, Turner H, Hamby S (2015). A revised inventory of adverse childhood experiences. Child Abus Negl.

[7] Houtepen LC, Heron J, Suderman MJ, Tilling K, Howe LD (2018). Adverse childhood experiences in the children of the Avon Longitudinal Study of Parents and Children (ALSPAC). Wellcome Open Res.

[8] Chandan JS, Thomas T, Gokhale KM, Bandyopadhyay S, Taylor J, Nirantharakumar K (2019). The burden of mental ill health associated with childhood maltreatment in the UK, using The Health Improvement Network database: a population-based retrospective cohort study. Lancet Psychiatry.

[9] Hughes K, Bellis MA, Hardcastle KA, Sethi D, Butchart A, Mikton C (2017). The effect of multiple adverse childhood experiences on health: a systematic review and meta-analysis. Lancet Public Health.

[10] Lupien SJ, McEwen BS, Gunnar MR, Heim C (2009). Effects of stress throughout the lifespan on the brain, behaviour and cognition. Nat Rev Neurosci.

[11] Koss KJ, Gunnar MR (2018). Annual Research Review: early adversity, the hypothalamic–pituitary–adrenocortical axis, and child psychopathology. J Child Psychol Psychiatry.

[12] Danese A, McEwen BS (2012). Adverse childhood experiences, allostasis, allostatic load, and age-related disease. Physiol Behav.

[13] Kuhlman KR, Chiang JJ, Horn S, Bower JE (2017). Developmental psychoneuroendocrine and psychoneuroimmune pathways from childhood adversity to disease. Neurosci Biobehav Rev.

[14] Carrion VG, Weems CF, Ray RD, Glaser B, Hessl D, Reiss AL (2002). Diurnal salivary cortisol in pediatric posttraumatic stress disorder. Biol Psychiatry.

[15] Gunnar MR, Morison SJ, Chisholm K, Schuder M (2001). Salivary cortisol levels in children adopted from Romanian orphanages. Dev Psychopathol.

[16] Hunter AL, Minnis H, Wilson P (2011). Altered stress responses in children exposed to early adversity: a systematic review of salivary cortisol studies. Stress.

[17] Chiang JJ, Taylor SE, Bower JE (2015). Early adversity, neural development, and inflammation. Dev Psychobiol.

[18] Bunea IM, Szentágotai-Tătar A, Miu AC (2017). Early-life adversity and cortisol response to social stress: a meta-analysis. Transl Psychiatry.

[19] Bernard K, Frost A, Bennett CB, Lindhiem O (2017). Maltreatment and diurnal cortisol regulation: a meta-analysis. Psychoneuroendocrinology.

[20] Khoury JE, Bosquet Enlow M, Plamondon A, Lyons-Ruth K (2019). The association between adversity and hair cortisol levels in humans: a meta-analysis. Psychoneuroendocrinology.

[21] Ouellet-Morin I et al. Associations between developmental trajectories of peer victimization, hair cortisol, and depressive symptoms: a longitudinal study. J Child Psychol Psychiatry 2020. 10.1111/jcpp.13228. 10.1111/jcpp.13228 32196669

[22] Stetler C, Miller GE (2011). Depression and hypothalamic-pituitary-adrenal activation: a quantitative summary of four decades of research. Psychosom. Med.

[23] Wilkinson PO, Goodyer IM (2011). Childhood adversity and allostatic overload of the hypothalamic-pituitary-adrenal axis: a vulnerability model for depressive disorders. Dev Psychopathol.

[24] Pan X, Wang Z, Wu X, Wen SW, Liu A (2018). Salivary cortisol in post-traumatic stress disorder: a systematic review and meta-analysis. BMC Psychiatry.

[25] Morris MC, Compas BE, Garber J (2012). Relations among posttraumatic stress disorder, comorbid major depression, and HPA function: a systematic review and meta-analysis. Clin Psychol Rev.

[26] Bosch NM, Riese H, Reijneveld SA, Bakker MP, Verhulst FC, Ormel J (2012). Timing matters: long term effects of adversities from prenatal period up to adolescence on adolescents’ cortisol stress response. The TRAILS study. Psychoneuroendocrinology.

[27] White LO, Ising M, von Klitzing K, Sierau S, Michel A, Klein AM (2017). Reduced hair cortisol after maltreatment mediates externalizing symptoms in middle childhood and adolescence. J Child Psychol. Psychiatry Allied Discip.

[28] Smoller JW, Andreassen OA, Edenberg HJ, Faraone SV, Glatt SJ, Kendler KS (2019). Psychiatric genetics and the structure of psychopathology. Mol Psychiatry.

[29] Ouellet-Morin I, Brendgen M, Girard A, Lupien SJ, Dionne G, Vitaro F (2016). Evidence of a unique and common genetic etiology between the CAR and the remaining part of the diurnal cycle: a study of 14 year-old twins. Psychoneuroendocrinology.

[30] Steptoe A, van Jaarsveld CHM, Semmler C, Plomin R, Wardle J (2009). Heritability of daytime cortisol levels and cortisol reactivity in children. Psychoneuroendocrinology.

[31] Jaffee SR, Price TS (2008). Genotype–environment correlations: implications for determining the relationship between environmental exposures and psychiatric illness. Psychiatry.

[32] Sheridan MA, McLaughlin KA (2014). Dimensions of early experience and neural development: deprivation and threat. Trends Cogn Sci.

[33] Kuhlman KR, Geiss EG, Vargas I, Lopez-Duran NL (2015). Differential associations between childhood trauma subtypes and adolescent HPA-axis functioning. Psychoneuroendocrinology.

[34] Busso DS, McLaughlin KA, Sheridan MA (2017). Dimensions of adversity, physiological reactivity, and externalizing psychopathology in adolescence: deprivation and threat. Psychosom Med.

[35] LoPilato AM (2020). Stress perception following childhood adversity: unique associations with adversity type and sex. Dev Psychopathol.

[36] Lacey, Minnis H (2020). Practitioner review: twenty years of adverse childhood experience (ACE) score research: strengths, limitations and application to practice. J Child Psychol Psychiatry.

[37] Haworth CMA, Davis OSP, Plomin R (2013). Twins Early Development Study (TEDS): a genetically sensitive investigation of cognitive and behavioral development from childhood to young adulthood. Twin Res Hum Genet.

[38] Ouellet-Morin I, Danese A, Bowes L, Shakoor S, Ambler A, Pariante CM (2011). A discordant monozygotic twin design shows blunted cortisol reactivity among bullied children. J Am Acad Child Adolesc Psychiatry.

[39] Khoury JE, Gonzalez A, Levitan RD, Pruessner JC, Chopra K, Basile VS (2015). Summary cortisol reactivity indicators: Interrelations and meaning. Neurobiol Stress.

[40] Angold A, Costello E, Pickles A, Winder F, Silver D (1987). The development of a questionnaire for use in epidemiological studies of depression in children and adolescents. Int J Methods Psychiatr Res.

[41] Waszczuk MA, Zavos HMS, Eley TC (2020). Why do depression, conduct, and hyperactivity symptoms co-occur across adolescence? The role of stable and dynamic genetic and environmental influences. Eur Child Adolesc Psychiatry.

[42] Baldwin JR, Arseneault L, Caspi A, Fisher HL, Moffitt TE, Odgers CL (2018). Childhood victimization and inflammation in young adulthood: a genetically sensitive cohort study. Brain Behav Immun.

[43] Brendgen M, Vitaro F, Bukowski WM, Dionne G, Tremblay RE, Boivin M (2013). Can friends protect genetically vulnerable children from depression?. Dev Psychopathol.

[44] Arseneault L, Cannon M, Fisher HL, Polanczyk G, Moffitt TE, Caspi A (2011). Childhood trauma and children’s emerging psychotic symptoms: a genetically sensitive longitudinal cohort study. Am J Psychiatry.

[45] Ravelle W psych: procedures for psychological, psychometric, and personality research. 2021. https://CRAN.R-project.org/package=psych.

[46] Rosseel Y (2012). lavaan: an R Package for Structural Equation Modeling. J Stat Softw.

[47] Jorgensen TD, Pornprasertmanit S, Schoemann AM, Rosseel Y semTools: Useful tools for structural equation modeling. R Packag version 05-4. https://CRAN.R-project.org/package=semTools.

[48] Linzer DA, Lewis JB (2011). poLCA: an R Package for Polytomous Variable Latent Class Analysis. J Stat Softw.

[49] Plomin R, DeFries JC, McClearn GE, McGuffin P. Behavioral Genetics, 5th edition. Worth, New Work: Cambridge University Press; 2008. 10.1375/twin.11.2.245 (accessed 2 Mar 2021).

[50] Bates TC, Maes H, Neale MC (2019). umx: Twin and Path-Based Structural Equation Modeling in R. Twin Res Hum Genet.

[51] Bates D, Mächler M, Bolker B, Walker S. Fitting linear mixed-effects models using lme4. J Stat Softw. 2015; **67**. 10.18637/jss.v067.i01.

[52] Tingley D, Yamamoto T, Hirose K, Keele L, Imai K (2014). Mediation: R package for causal mediation analysis. J Stat Softw.

[53] Buuren S van. Package ‘mice’. Multivariate Imputation by Chained Equations. 2019. 10.18637/jss.v045.i03.

[54] Kenny DA. Multiple Latent Variable Models: Confirmatory Factor Analysis. 2016. http://davidakenny.net/cm/mfactor.htm (accessed 22 May 2019).

[55] Field A. Discovering statistics using SPSS. SAGE Publications Limited: London, 2013.

[56] Kendler KS, Gatz M, Gardner CO, Pedersen NL (2006). A Swedish National Twin Study of Lifetime Major Depression. Am J Psychiatry.

[57] VanderWeele TJ, Ding P (2017). Sensitivity analysis in observational research: introducing the E-value. Ann Intern Med.

[58] Iob E, Kirschbaum C, Steptoe A (2020). Persistent depressive symptoms, HPA-axis hyperactivity, and inflammation: the role of cognitive-affective and somatic symptoms. Mol Psychiatry.

[59] Assary E, Vincent JP, Keers R, Pluess M (2018). Gene-environment interaction and psychiatric disorders: review and future directions. Semin Cell Dev Biol.

[60] LeMoult J, Humphreys KL, Tracy A, Hoffmeister JA, Ip E, Gotlib IH (2020). Meta-analysis: exposure to early life stress and risk for depression in childhood and adolescence. J. Am. Acad. Child Adolesc. Psychiatry.

[61] Humphreys KL, LeMoult J, Wear JG, Piersiak HA, Lee A, Gotlib IH (2020). Child maltreatment and depression: a meta-analysis of studies using the Childhood Trauma Questionnaire. Child Abus Negl.

[62] Infurna MR, Reichl C, Parzer P, Schimmenti A, Bifulco A, Kaess M (2016). Associations between depression and specific childhood experiences of abuse and neglect: a meta-analysis. J. Affect Disord.

[63] Nelson J, Klumparendt A, Doebler P, Ehring T (2017). Childhood maltreatment and characteristics of adult depression: meta-analysis. Br J Psychiatry.

[64] Baldwin JR, Reuben A, Newbury JB, Danese A (2019). Agreement between prospective and retrospective measures of childhood maltreatment. JAMA Psychiatry.

[65] Newbury JB, Arseneault L, Moffitt TE, Caspi A, Danese A, Baldwin JR (2018). Measuring childhood maltreatment to predict early-adult psychopathology: comparison of prospective informant-reports and retrospective self-reports. J Psychiatr Res.

[66] Reuben A, Moffitt TE, Caspi A, Belsky DW, Harrington H, Schroeder F (2016). Lest we forget: comparing retrospective and prospective assessments of adverse childhood experiences in the prediction of adult health. J Child Psychol Psychiatry.

[67] Ellis BJ, Abrams LS, Masten AS, Sternberg RJ, Tottenham N, Frankenhuis WE. Hidden talents in harsh environments. Dev Psychopathol. 2020;1–19. 10.1017/S0954579420000887 32672144

