# Supplementary material for: Adverse childhood experiences, daytime salivary cortisol, and depressive symptoms in early adulthood: a longitudinal genetically informed twin study
Source: Transl Psychiatry. 2021 Aug 5;11:420. doi: 10.1038/s41398-021-01538-w (PMC8342545; doi:10.1038/s41398-021-01538-w)
Supplement: Supplementary file 1 — Appendix file [file 41398_2021_1538_MOESM1_ESM.docx]

**Appendix**

**sMethods**

**ACEs measures**

Exposure to several ACEs was assessed repeatedly from age 3 to 11 years using both prospectively and retrospectively collected information, reported by the parents and the twins. These included: Negative Parental Practices, Negative Parental Feelings, Maternal Depression, Divorce of Parents, Separation from Parents, Bullying, Emotional Abuse, and Physical Abuse. **Negative parental practices** were assessed using the Parental Discipline scale, which was adapted from a semi-structured interview developed by Deater-Deckard *et al.* (1998)^1^. This scale includes six questions about different discipline methods used by parents. Each item was rated on a 5-point scale ranging from “never” to “usually”. The total score representing negative parental practices was calculated by subtracting the total score of positive parental practices (e.g. “Explain to child, or reason with child”) from that of negative practices (e.g. “Give a smack or slap”). The scale has been previously shown to have low internal consistency in the TEDS sample (α ~ 0.60)^2,3^. The Cronbach's α of this scale in our analytical sample was around 0.60 at all time points. We tried removing one or more items, but this only resulted in an increase of 0.01-0.03 points in the α value. Nevertheless, it has been suggested that α values between 0.60 and 0.70 can also be deemed acceptable, particularly if the scale has only few items and the sample size is small^4^, as in our study. In addition, this scale has been found to be associated with child psychopathology in previous TEDS studies^2,3^, thereby demonstrating predictive validity. **Negative parental feelings** were measured using the Parent Feelings Questionnaire^5^, which contains seven questions about different types of positive and negative feelings experienced by parents towards their children (e.g. “Feel impatient”, “Feel quite happy”, “She/he makes me angry”). Each item was scored on a 5-point scale ranging from “Very untrue” to “Very true”. The total score indicating negative parental feelings was calculated by subtracting the total positive feelings score from the negative feelings score. The internal consistency of this scale in the TEDS sample is good (α ~ 0.70)^3,6^. **Maternal Depression** was ascertained using the Edinburgh Postnatal Depression Scale (EPDS)^7^, a 10-item self-report scale screening for postnatal depressive symptoms among mothers (e.g. “I have blamed myself unnecessarily when things went wrong”, “I have been anxious or worried for no good reason”, “I have felt sad or miserable”). Each item was scored on a 4-point scale ranging from “No, not at all” to “Yes, most of the time”. The presence of depression was defined as EPDS total scores higher than 10 points. The scale has been validated against diagnostic criteria in the postpartum period, and has good internal validity in the TEDS sample (α ~ 0.70)^8^. Information on **Divorce** of parents and **Separation** from parents for a prolonged period of time was collected as part of a life events questionnaire. Both items were coded as binary variables (no/yes). **Bullying** was assessed using the Multidimensional Peer Victimisation Scale^9^. This measure includes 16 items which are divided into four subscales: physical bullying (e.g. “Kicked me”), verbal bullying (e.g. “Called me names”), social manipulation (e.g. “Tried to make my friends turn against me”), and property attacks (e.g. “Tried to break something of mine”). For each item, participants were asked how often they experienced the bullying event during the past year on a 3-point scale ranging from “not at all” to “more than once”. The total bullying score was calculated by taking the mean of the 4 subscales. This scale has been shown to have high internal validity in the TEDS sample (α ~ 0.85)^10^. **Physical and emotional abuse** were assessed through a retrospective questionnaire about negative childhood experiences that happened before the age of 11 years, which was completed by the twins at age 21 years. The questionnaire includes 4 questions about emotional abuse (e.g. “Say hurtful or insulting things to you”) and other 4 questions about physical abuse (e.g. “Push, grab or shove you”) by a family member. Each item was scored on a 5-point scale ranging from “Never” to “Very often”, and the total score was calculated by adding together the item scores for each type of abuse. Both scales demonstrate high internal validity in our sample (Emotional abuse: α = 0.83; Physical abuse: α = 0.78).

**Deriving data-driven ACEs clusters**

In order to identify distinct ACEs clusters underlying the individual ACE indicators we applied both FA (variable-centred method) and LCA (i.e. person-centred method) clustering techniques.

*FA-derived ACEs clusters*

FA was employed to derive ACEs clusters representing groups of ACEs that are most likely to co-occur in the sample. We used a combination of explorative factor analysis (EFA) and confirmatory factor analysis (CFA) with 2-fold cross-validation, which can help to reduce the risk of overfitting, as described elsewhere^11^. After randomly splitting the sample into training and test data (50/50 split), EFA with geomin rotation was conducted in the training dataset to establish the number of latent factors and their item loadings. This process was informed by inspection of scree plots and eigenvalues. We then performed CFA on the test dataset, using the EFA results to specify the number of latent factors and item loadings. The CFA model was tested using a diagonally weighted least squares (DWLS) estimator, which is specifically designed for categorical data and has been shown to outperform ML in estimating factor loadings across different conditions^12^. Model fit was evaluated using Standardised Root Mean Square Residual (SRMSR), Root Mean Square Error of Approximation (RMSEA), Comparative Fit Index (CFI), and Tucker Lewis Index (TLI). RMSEA and SRMSR are measures of absolute fit which should be smaller than .08. CFI and TLI are comparative fit indices which should be greater than .90^13^.

*LCA-derived ACEs clusters*

LCA was employed to derive person-centred ACEs clusters representing classes of participants co-reporting similar ACEs. Models for 2 to 6 classes were compared in both the training and test datasets to check whether the same best-fitting class solution emerged in both samples. The best fitting class solution was determined by comparing the model fit indices Akaike’s Information Criteria (AIC) and Bayesian Information Criteria (BIC). Lower values of the AIC and BIC indicate a better fitting model. In deciding on the best fitting model we gave preference to improvements in the BIC, as recommended elsewhere^14^. The predicted probabilities of each ACE in each class and a latent profile plot for the final class solution were used to aid with the qualitative interpretation of the derived classes.

**Mediation analysis**

We performed model-based causal mediation analysis using the *Mediation* R package^15^ to examine whether the exposure (ACEs) was indirectly associated with the outcome (depressive symptoms) through the mediator (cortisol) (Figure1, Manuscript). This mediation analysis approach is embedded within the counterfactual framework of causal inference and does not make reference to any specific statistical model. Consequently, it can be applied to a wide range of data settings including mixed-effects models. The average causal mediation effect (ACME) (or indirect effect) is defined as follow:

﻿*δ_i_ (e) = ﻿ ﻿Y_i_(e,M_i_(1)) −Y_i_(e,M_i_(0)),*

﻿for each unit *i* and each exposure status *e* = 0, 1. This equation represents the change in the outcome that would occur by changing the mediator *(M)* from the value that would result under the unexposed condition *M_i_(0)* to the value that would be realised under the exposed condition *M_i_(1)*, while holding exposure status constant^16^. Model-based ACMEs are estimated under the sequential ﻿ignorability assumption (i.e. no unmeasured confounders affecting the relationship of the exposure with the mediator and the outcome, and the relationship between the mediator and the outcome), which can be satisfied by including the relevant confounding variables in the mediation models. Causal mediation analysis also allows for the exploration of whether the ACME varies depending on the baseline exposure status. This can be tested by adding an interaction term between the exposure and the mediator to the outcome model, which provides the ACME under the exposed and unexposed conditions.

**Multiple imputation**

Missing data on the outcome and exposure variables were estimated using multiple imputation by chained equations (MICE) with the *mice* R package^17^. The proportions of missing data for all imputed variables are shown in sTable9. The imputation models included all study variables. We generated 20 imputed datasets and combined all estimates from regression and causal mediation analysis using Rubin’s rules^18^.

**sResults**

**Descriptive statistics**

Children were aged 11 years on average at the cortisol assessment, and 60% of the sample was female. One third of the participants had high family SES. Around 30% of children reported high (i.e. top tertile) levels of emotional or physical abuse from parents, and another 30% reported high exposure to bullying by peers. 12% of children experienced parental divorce or were separated from their parents for a prolonged period of time. In addition, almost two thirds of the participants reported dysfunctional parenting experiences. The mean value of average cortisol across the three assessments was 3.47 nmol/l. On average, cortisol levels decreased from baseline to post-task 1 (mean change = -0.632 nmol/l), suggesting that the study protocol did not elicit increases in cortisol responses. The average levels of depressive symptoms in the sample were generally low (average total score = 4), but ranged across the full spectrum from no symptoms to severe symptoms.

**References**

1 Deater-Deckard K, Dodge KA, Bates JE, Pettit GS. Multiple risk factors in the development of externalizing behavior problems: Group and individual differences. *Dev Psychopathol* 1998; **10**: 469–493.

2 Viding E, Fontaine NMG, Oliver BR, Plomin R. Negative parental discipline, conduct problems and callous-unemotional traits: Monozygotic twin differences study. *Br J Psychiatry* 2009; **195**: 414–419.

3 Wilkinson PO, Trzaskowski M, Haworth CMA, Eley TC. The role of gene-environment correlations and interactions in middle childhood depressive symptoms. *Dev Psychopathol* 2013; **25**: 93–104.

4 Hair J, Black W, Babin B, Anderson R, Tatham R. *ultivariate Data Analysis (6th ed.)*. Pearson Educational, Inc.: New Jersey, 2006.

5 Deater-Deckard K. Parenting and Child Behavioral Adjustment in Early Childhood: A Quantitative Genetic Approach to Studying Family Processes. *Child Dev* 2000; **71**: 468–484.

6 Hannigan LJ, McAdams TA, Eley TC. Developmental change in the association between adolescent depressive symptoms and the home environment: results from a longitudinal, genetically informative investigation. *J Child Psychol Psychiatry* 2017; **58**: 787–797.

7 Cox JL, Holden JM, Sagovsky R. Detection of Postnatal Depression: Development of the 10-item Edinburgh Postnatal Depression scale. *Br J Psychiatry* 1987; **150**: 782–786.

8 Asbury K, Dunn JF, Pike A, Plomin R. Nonshared Environmental Influences on Individual Differences in Early Behavioral Development: A Monozygotic Twin Differences Study. *Child Dev* 2003; **74**: 933–943.

9 Mynard H, Joseph S. Development of the multidimensional peer-victimization scale. *Aggress Behav* 2000; **26**: 169–178.

10 Fontaine NMG, Hanscombe KB, Berg MT, McCrory EJ, Viding E. Trajectories of Callous-Unemotional Traits in Childhood Predict Different Forms of Peer Victimization in Adolescence. *J Clin Child Adolesc Psychol* 2018; **47**: 458–466.

11 Iob E, Lacey R, Steptoe A. The long-term association of adverse childhood experiences with C-reactive protein and hair cortisol: Cumulative risk versus dimensions of adversity. *Brain Behav Immun* 2019. doi:10.1016/j.bbi.2019.12.019.

12 Li C-H. Confirmatory factor analysis with ordinal data: Comparing robust maximum likelihood and diagonally weighted least squares. *Behav Res Methods* 2016. doi:10.3758/s13428-015-0619-7.

13 Byrne BM. *Structural equation modeling with Mplus: Basic concepts, applications, and programming.* Routledge/Taylor & Francis Group: New York, US, 2012.

14 Nylund-Gibson K, Young Choi A. Ten frequently asked questions about latent class analysis. *Transl Issues Psychol Sci* 2018; **4**: 440–461.

15 Tingley D, Yamamoto T, Hirose K, Keele L, Imai K. Mediation: R package for causal mediation analysis. *J Stat Softw* 2014; **59**: 1–38.

16 Imai K, Keele L, Tingley D. A General Approach to Causal Mediation Analysis. *Psychol Methods* 2010; **15**: 309–334.

17 Buuren S van. Package ‘mice’. Multivariate Imputation by Chained Equations. 2019. doi:10.18637/jss.v045.i03.

18 Rubin DB. *Multiple Imputation for Nonresponse in Surveys*. John Wiley and Sons: New York, 2004.

19 VanderWeele TJ, Ding P. Sensitivity Analysis in Observational Research: Introducing the E-Value. *Ann Intern Med* 2017; **167**: 268–274.

20 Field A. *Discovering statistics using SPSS*. SAGE Publications Limited: London, 2013.

**Supplementary tables and figures**

| **sTable 1. Comparison of baseline characteristics between the analytical sample and the full TEDS sample.** | | | | |
| --- | --- | --- | --- | --- |
|  | Excluded families (N=13509) | Included families  (N=145) | P-value comparison | Correlation (r) |
| **Sex - twin 1** |  |  | 0.051 | -0.017 |
| N-Miss | 1 | 0 |  |  |
| Mean (SD) | 0.504 (0.500) | 0.437 (0.497) |  |  |
| **Sex - twin 2** |  |  | 0.084 | -0.015 |
| N-Miss | 1 | 0 |  |  |
| Mean (SD) | 0.496 (0.500) | 0.437 (0.497) |  |  |
| **Ethnicity** |  |  | 0.676 | 0.004 |
| N-Miss | 53 | 0 |  |  |
| Mean (SD) | 1.917 (0.276) | 1.925 (0.264) |  |  |
| **Mother's higher education** | |  | 0.238 | -0.010 |
| N-Miss | 291 | 4 |  |  |
| Mean (SD) | 1.355 (0.479) | 1.316 (0.466) |  |  |
| **Mother's social class** | |  | 0.308 | 0.013 |
| N-Miss | 7848 | 124 |  |  |
| Mean (SD) | 2.855 (1.234) | 2.989 (1.039) |  |  |
| **Maternal smoking during pregnancy** | |  | 0.775 | -0.002 |
| N-Miss | 199 | 2 |  |  |
| Mean (SD) | 0.183 (0.387) | 0.175 (0.381) |  |  |
| **High maternal stress during pregnancy** | |  | 0.235 | -0.010 |
| N-Miss | 91 | 1 |  |  |
| Mean (SD) | 1.218 (0.413) | 1.184 (0.388) |  |  |
| **Note.** *p-values from significance tests including t-tests (continuous variables) or chi-square tests (categorical variables). r coefficients obtained from Spearman’s Rho correlation tests. N-Miss = number of missing values. SD = standard deviation.  Sex: 0=female;1=male. Ethnicity: 1=non-white; 2=white. Mother's higher education: 1=no; 2=yes. 1(min)=lowest; 6(max)=highest. Maternal smoking: 0=No; 1=yes. High maternal stress: 1=No; 2=Yes. | | | | |

| **sTable 2. Parental Discipline scale.** |
| --- |
| **Negative discipline methods**   1. Give a smack or slap 2. Telling off or shouting 3. Make a joke out of it 4. Ask someone else to deal with the situation (for example, other parent)   **Positive discipline methods**   1. Explain to child, or reason with child 2. Be firm and calm with child |

| **sTable 3. Parent Feelings Questionnaire.** |
| --- |
| **Negative feelings**   1. Sometimes I feel very impatient with him/her 2. Sometimes I wish s/he would go away for a few minutes 3. Sometimes s/he makes me angry 4. Sometimes I am frustrated by him/her   **Positive feelings**   1. I usually feel quite happy about my relationship with him/her 2. Sometimes I am amused by him/her 3. I usually feel close to him/her |
| **Note.** Questions included in the 3 Year Parent Questionnaire. Slightly modified items were used in the following assessments. |

| **sTable 4. Edinburgh Postnatal Depression Scale (EPDS).** |
| --- |
| 1. I have been able to laugh and see the funny side of things* 2. I have looked forward with enjoyment to things* 3. I have blamed myself unnecessarily when things went wrong 4. I have been anxious or worried for no good reason 5. I have felt scared or panicky for no very good reason 6. Things have been getting on top of me 7. I have been so unhappy that I have had difficulty sleeping 8. I have felt sad or miserable 9. I have been so unhappy that I have been crying 10. The thought of harming myself has occurred to me |
| **Note.** *Positive items were reverse scored to calculate the total EPDS score. |

| **sTable 5. Multidimensional Peer Victimisation Scale.** |
| --- |
| “How often during this school year has another pupil done these things to you?”   1. Punched me 2. Tried to get me into trouble with my friends 3. Called me names 4. Took something of mine without permission 5. Kicked me 6. Tried to make my friends turn against me 7. Made fun of me because of my appearance 8. Tried to break something of mine 9. Hurt me physically in some way 10. Refused to talk to me 11. Made fun of me for some reason 12. Stole something from me 13. Beat me up 14. Made other people not talk to me 15. Swore at me 16. Deliberately damaged some property of mine |

| **sTable 6. Negative Childhood Experiences Questionnaire.** |
| --- |
| “Before the age of 11, how often did an adult in your family:”  **Emotional abuse**   1. Shout at you 2. Say hurtful or insulting things to you 3. Punish you in a way that seemed cruel 4. **Threaten** to kick, punch, or hit you with something that could hurt you or physically attack you in another way   **Physical abuse**   1. Push, grab or shove you 2. Smack you for discipline 3. **Actually** kick, punch, or hit you with something that could hurt you or physically attack you in another way 4. Hit you so hard it left you with bruises or marks |

| **sTable 7. Time points, sources, and methods of data collection for each type of ACE.** | | | |
| --- | --- | --- | --- |
| **ACE measure** | **Time points** | **Source** | **Method** |
| Negative parental practices | 3, 4, 7, 9, and 11 years | Parent-reported: 3-11 years; Child-reported: 11 years. | Prospective |
| Negative parental feelings | 3, 4, 7, 9, and 11 years | Parent-reported: 3-11 years; Child-reported: 11 years. | Prospective |
| Maternal depression | 3 and 4 years | Parent-reported | Prospective |
| Divorce of parents | 7 and 9 years | Parent-reported | Prospective |
| Separation from parents | 7 and 9 years | Parent-reported | Prospective |
| Bullying | 11 years | Child-reported | Prospective |
| Emotional abuse | 21 years | Child-reported | Retrospective |
| Physical abuse | 21 years | Child-reported | Retrospective |

| **sTable 8. Short Mood and Feeling Questionnaire.** |
| --- |
| “In the past two weeks…”   1. I felt miserable or unhappy 2. I felt so tired I just sat around and did nothing 3. I was very restless 4. I cried a lot 5. I found it hard to think properly or concentrate 6. I hated myself 7. I felt lonely 8. I thought I could never be as good as other people |

| **sTable 9. Characteristics of the study participants and comparison with imputed data.** | | | | | | |
| --- | --- | --- | --- | --- | --- | --- |
|  | **Observed data** | | | **Imputed data*** | | |
|  | **MZ twins (N=159)** | **DZ twins (N=131)** | **Total (N=290)** | **MZ twins (N=159)** | **DZ twins (N=131)** | **Total (N=290)** |
| **Sex** |  |  |  |  |  |  |
| Girls | 101 (63.5%) | 72 (55.0%) | 173 (59.7%) | 63.50% | 55.00% | 59.70% |
| Boys | 58 (36.5%) | 59 (45.0%) | 117 (40.3%) | 36.50% | 45.00% | 40.30% |
| **Age in years** |  |  |  |  |  |  |
| Mean (SD) | 11.07 (0.57) | 11.25 (0.45) | 11.15 (0.52) | 11.07 (0.57) | 11.25 (0.44) | 11.15 (0.52) |
| Range | 9.90 - 12.30 | 10.19 - 12.19 | 9.90 - 12.30 | 9.90 - 12.30 | 10.19 -12.19 | 9.90 - 12.30 |
| **Family SES** |  |  |  |  |  |  |
| N-Miss/ % | 4 / 2.5% | 2 / 2% | 6 / 2% |  |  |  |
| High | 54 (34.8%) | 40 (31.0%) | 94 (33.1%) | 34.60% | 31.10% | 33.00% |
| **ACEs cumulative score** | |  |  |  |  |  |
| 0 ACEs | 21 (30.9%) | 10 (27.0%) | 31 (29.5%) | 25.80% | 22.10% | 24.10% |
| 1 ACE | 18 (26.5%) | 9 (24.3%) | 27 (25.7%) | 23.00% | 25.60% | 24.20% |
| 2 ACEs | 15 (22.1%) | 5 (13.5%) | 20 (19.0%) | 20.80% | 19.00% | 20.00% |
| 3+ ACEs | 14 (20.6%) | 13 (35.1%) | 27 (25.7%) | 30.40% | 33.30% | 31.70% |
| **Average cortisol (age 11), ﻿nmol/l** | |  |  |  |  |  |
| Mean (SD) | 3.32 (1.66) | 3.64(1.89) | 3.46 (1.77) | 3.31 (1.65) | 3.64 (1.87) | 3.46 (1.76) |
| Range | 0.14 - 10.11 | 0.50 - 9.10 | 0.14 - 10.11 | 0.14 - 10.11 | 0.50 - 9.10 | 0.14 - 10.11 |
| **Cortisol reactivity (age 11), ﻿nmol/l** | |  |  |  |  |  |
| Mean (SD) | -0.55 (1.16) | -0.72 (1.53) | -0.63 (1.34) | -0.56 (1.15) | -0.72 (1.52) | -0.63 (1.34) |
| Range | -5.63 - 3.06 | -6.23 - 4.53 | -6.23 - 4.53 | -5.63 - 3.06 | -6.23 - 4.53 | -6.23 - 4.53 |
| **Depressive symptoms (age 21)** | |  |  |  |  |  |
| N-Miss / % | 60 / 37% | 66 / 50% | 126 / 43% |  |  |  |
| Mean (SD) | 4.97 (4.58) | 4.12 (4.25) | 4.63 (4.46) | 4.93 (4.63) | 4.21 (4.33) | 4.60 (4.52) |
| Range | 0.00 - 16.00 | 0.00 - 15.00 | 0.00 - 16.00 | 0.00 - 16.00 | 0.00 - 16.00 | 0.00 - 16.00 |
| ***Note.*** MZ = Monozygotic; DZ = Dizygotic; SES = socioeconomic status; ACEs = Adverse childhood experiences; N-Miss = number of missing data; SD = standard deviation.  *Only percentages are reported for the imputed data as counts vary across the 20 imputed datasets. | | | | | | |

| **sTable 10. Prevalence of the ACEs items in the sample.** | | |
| --- | --- | --- |
|  | **Observed data (N=290)** | **Imputed data*** |
| **Negative Parental Practices** |  |  |
| N-Miss | 119 |  |
| No | 114 (66.7%) | 63.70% |
| Yes | 57 (33.3%) | 36.30% |
| **Negative Parental Feelings** |  |  |
| N-Miss | 118 |  |
| No | 115 (66.9%) | 63.10% |
| Yes | 57 (33.1%) | 36.90% |
| **Maternal Depression** |  |  |
| N-Miss | 18 |  |
| No | 180 (66.2%) | 66.00% |
| Yes | 92 (33.8%) | 34.00% |
| **Divorce** |  |  |
| N-Miss | 71 |  |
| No | 200 (91.3%) | 89.70% |
| Yes | 19 (8.7%) | 10.30% |
| **Separation** |  |  |
| N-Miss | 80 |  |
| No | 200 (95.2%) | 91.50% |
| Yes | 10 (4.8%) | 8.50% |
| **Bullying** |  |  |
| N-Miss | 53 |  |
| No | 159 (67.1%) | 66.50% |
| Yes | 78 (32.9%) | 33.50% |
| **Emotional Abuse** |  |  |
| N-Miss | 136 |  |
| No | 114 (74.0%) | 69.40% |
| Yes | 40 (26.0%) | 30.60% |
| **Physical Abuse** |  |  |
| N-Miss | 137 |  |
| No | 144 (94.1%) | 89.70% |
| Yes | 9 (5.9%) | 10.30% |
| ***Note.*** ACEs = Adverse childhood experiences; N-Miss = number of missing data; *Only percentages are reported for the imputed data as counts vary across the 20 imputed datasets. | | |

| **sTable 11. Explorative factor analysis (EFA) of the ACE binary indicators in the training data.** | | | |
| --- | --- | --- | --- |
| **ACE items** | **Negative Parental Experiences**  **(Factor 1)** | **Divorce/ Separation**  **(Factor 2)** | **Abuse**  **(Factor 3)** |
| Negative Parental Practices | **0.501** | -0.019 | 0.148 |
| Negative Parental Feelings | **0.748** | 0.011 | -0.047 |
| Maternal Depression | **0.493** | 0.000 | 0.012 |
| Divorce | -0.003 | **0.999** | -0.008 |
| Separation | 0.073 | **0.373** | 0.169 |
| Bullying | 0.202 | -0.097 | 0.043 |
| Emotional Abuse | 0.015 | 0.006 | **0.672** |
| Physical Abuse | -0.031 | -0.015 | **0.536** |
| ***Note.*** Bold coefficients indicate the highest factor loading of every item. According to Field (2013)^20^, items with factor loadings less than 0.3 should be removed. Bullying had factor loadings less than 0.3 on all factors, and was therefore included as a standalone dimension in the CFA model performed in the test dataset. | | | |

| **sTable 12. Measurement invariance of the final CFA model across the training and test datasets.** | | | | | |
| --- | --- | --- | --- | --- | --- |
|  | **AIC** | **BIC** | **Chisq** | **Chisq diff** | **p-value^a^** |
| Model 1: configural invariance | 41669 | 42056 | 426.26 |  |  |
| Model 2: weak invariance | 41666 | 42026 | 431.48 | 5.2276 | 0.2647 |
| Model 3: strong invariance | 41664 | 41991 | 439.55 | 8.0674 | 0.1526 |
| ***Note.*** Measurement invariance was established by comparing a model with the same factor structure imposed on both datasets (Model 1; configural invariance) to a model where the factor loadings are constrained to be equal across the datasets (Model 2; weak invariance), and a second model where both factor loadings and intercepts are constrained to be equal across the datasets (Model 3; strong invariance). Weak/strong measurement invariance is supported if the p-value of the test comparing Model 2/3 vs Model 1 is non-significant. | | | | | |

| **sTable 13. Model fit of the 2 to 6 classes LCA models in the training data, test data, and whole sample.** | | | | | |
| --- | --- | --- | --- | --- | --- |
|  | **2 Classes** | **3 Classes** | **4 Classes** | **5 Classes** | **6 Classes** |
| ***Training data*** |  |  |  |  |  |
| AIC | 22563.83 | 22269.37 | 22178.25 | **21955.32** | 21930.27 |
| BIC | 22665.36 | 22424.65 | 22387.29 | **22218.11** | 22246.81 |
| ***Test data*** |  |  |  |  |  |
| AIC | 22951.39 | 22770.83 | 22498.80 | **22388.46** | 22348.90 |
| BIC | 23052.93 | 22926.12 | 22707.84 | **22651.25** | 22665.44 |
| ***Whole sample*** |  |  |  |  |  |
| AIC | 46224.94 | 45786.31 | 45228.59 | **45011.63** | 44965.30 |
| BIC | 46338.57 | 45960.09 | 45462.51 | **45305.70** | 45319.53 |

**sFigure 1. ACE twin model results.**

***Note.*** Additive genetic (A), shared environment (C) and non-shared environment (E) effects and 95% confidence intervals.

| **sTable 14. Associations of ACEs with average daytime cortisol levels (age 11).** | | | | | | | | |
| --- | --- | --- | --- | --- | --- | --- | --- | --- |
|  | **Model** | **Estimate**  **(b)** | **SE** | **P-value** | **Lower CI** | **Upper CI** | **Effect size** | **E-value** |
| **ACEs cumulative score (ref: 0 ACEs)** | | | |  |  |  |  |  |
| 1 ACE | Model 1 | -0.091 | 0.346 | 0.792 | -0.770 | 0.587 | -0.009 |  |
|  | Model 2 | -0.095 | 0.318 | 0.764 | -0.718 | 0.527 | -0.010 |  |
| 2 ACEs | Model 1 | -0.124 | 0.392 | 0.752 | -0.893 | 0.645 | -0.012 |  |
|  | Model 2 | -0.026 | 0.357 | 0.943 | -0.726 | 0.675 | -0.003 |  |
| 3+ ACEs | Model 1 | **-0.576** | **0.291** | **0.049** | **-1.147** | **-0.005** | **-0.066** | **1.768** |
|  | Model 2 | -0.407 | 0.249 | 0.100 | -0.895 | 0.080 | -0.045 |  |
| **FA-derived ACEs clusters** | | | |  |  |  |  |  |
| Abuse | Model 1 | -0.334 | 0.347 | 0.337 | -1.015 | 0.347 | -0.033 |  |
|  | Model 2 | -0.067 | 0.311 | 0.830 | -0.676 | 0.542 | -0.007 |  |
| Bullying | Model 1 | **-0.611** | **0.227** | **0.008** | **-1.056** | **-0.166** | **-0.061** |  |
|  | Model 2 | **-0.510** | **0.204** | **0.013** | **-0.910** | **-0.111** | **-0.051** | **1.924** |
| Separation/  Divorce | Model 1 | 0.177 | 0.401 | 0.660 | -0.610 | 0.963 | 0.018 |  |
|  | Model 2 | 0.194 | 0.340 | 0.569 | -0.472 | 0.859 | 0.019 |  |
| Dysfunctional parenting | Model 1 | -0.152 | 0.277 | 0.585 | -0.696 | 0.392 | -0.015 |  |
|  | Model 2 | -0.105 | 0.235 | 0.655 | -0.565 | 0.355 | -0.011 |  |
| **LCA-derived ACEs clusters (ref: Low ACEs)** | | | |  |  |  |  |  |
| Dysfunctional parenting/  Emotional abuse | Model 1 | -0.405 | 0.399 | 0.312 | -1.187 | 0.378 | -0.041 |  |
|  | Model 2 | -0.122 | 0.354 | 0.731 | -0.817 | 0.572 | -0.012 |  |
| Dysfunctional parenting | Model 1 | -0.051 | 0.273 | 0.851 | -0.586 | 0.483 | -0.005 |  |
|  | Model 2 | -0.068 | 0.237 | 0.774 | -0.533 | 0.397 | -0.007 |  |
| Separation/  Divorce | Model 1 | 0.635 | 0.502 | 0.207 | -0.350 | 1.620 | 0.064 |  |
|  | Model 2 | 0.485 | 0.434 | 0.265 | -0.365 | 1.335 | 0.049 |  |
| Emotional abuse | Model 1 | **-0.844** | **0.347** | **0.016** | **-1.524** | **-0.165** | **-0.085** | **2.459** |
|  | Model 2 | -0.470 | 0.312 | 0.133 | -1.080 | 0.141 | -0.047 |  |
| ***Note****.* N=290. Pooled estimates from mixed-effects linear regression analysis with 20 imputed datasets. Model 1 = adjusted for sex, age, and family SES; Model 2 = Model 1 + latent genetic liability for cortisol. Values in bold represent statistically significant coefficients at the 5% level (p=<0.05). SE = standard error. CI = 95% confidence interval. | | | | | | | | |

| **sTable 15. Associations of ACEs and average daytime cortisol levels (age 11) with depressive symptoms (age 21).** | | | | | | | | |
| --- | --- | --- | --- | --- | --- | --- | --- | --- |
|  | **Model** | **Estimate**  **(b)** | **SE** | **P-value** | **Lower CI** | **Upper CI** | **Effect size** | **E-value** |
| **Cortisol** | Model 1 | -0.322 | 0.181 | 0.079 | -0.677 | 0.034 | -0.020 |  |
|  | Model 2 | -0.242 | 0.133 | 0.072 | -0.503 | 0.020 | -0.013 |  |
| **ACEs cumulative score (ref: 0 ACEs)** | | | |  |  |  |  |  |
| 1 ACE | Model 1 | 0.864 | 0.907 | 0.343 | -0.914 | 2.643 | 0.043 |  |
|  | Model 2 | 0.984 | 0.599 | 0.103 | -0.190 | 2.158 | 0.050 |  |
| 2 ACEs | Model 1 | 0.948 | 0.987 | 0.340 | -0.987 | 2.882 | 0.070 |  |
|  | Model 2 | 0.914 | 0.665 | 0.172 | -0.390 | 2.217 | 0.063 |  |
| 3+ ACEs | Model 1 | **2.368** | **0.941** | **0.014** | **0.524** | **4.211** | **0.194** |  |
|  | Model 2 | **1.601** | **0.587** | **0.007** | **0.451** | **2.750** | **0.132** | **2.349** |
| **FA-derived ACEs clusters** | |  |  |  |  |  |  |  |
| Abuse | Model 1 | **2.114** | **0.962** | **0.031** | **0.228** | **4.000** | **0.132** |  |
|  | Model 2 | **1.537** | **0.635** | **0.017** | **0.293** | **2.781** | **0.096** | **2.029** |
| Bullying | Model 1 | **1.860** | **0.854** | **0.032** | **0.186** | **3.534** | **0.116** |  |
|  | Model 2 | **1.047** | **0.473** | **0.030** | **0.119** | **1.974** | **0.065** | **1.698** |
| Separation/  Divorce | Model 1 | -0.006 | 1.356 | 0.996 | -2.664 | 2.652 | 0.000 |  |
|  | Model 2 | -0.035 | 0.790 | 0.965 | -1.584 | 1.514 | -0.002 |  |
| Dysfunctional parenting | Model 1 | 0.952 | 0.697 | 0.175 | -0.413 | 2.318 | 0.060 |  |
|  | Model 2 | 0.703 | 0.479 | 0.145 | -0.236 | 1.642 | 0.044 |  |
| **LCA-derived ACEs clusters (ref: Low ACEs)** | | | | |  |  |  |  |
| Dysfunctional parenting/  Emotional Abuse | Model 1 | **2.415** | **1.114** | **0.032** | **0.231** | **4.600** | **0.151** |  |
|  | Model 2 | **1.749** | **0.736** | **0.019** | **0.307** | **3.191** | **0.109** | **2.196** |
| Dysfunctional parenting | Model 1 | 0.992 | 0.773 | 0.202 | -0.523 | 2.506 | 0.062 |  |
|  | Model 2 | 0.766 | 0.509 | 0.135 | -0.231 | 1.763 | 0.048 |  |
| Separation/Divorce | Model 1 | -0.935 | 1.670 | 0.578 | -4.208 | 2.338 | -0.058 |  |
|  | Model 2 | -0.866 | 0.928 | 0.353 | -2.684 | 0.952 | -0.054 |  |
| Emotional abuse | Model 1 | 1.532 | 1.085 | 0.161 | -0.595 | 3.659 | 0.096 |  |
|  | Model 2 | **1.949** | **0.671** | **0.004** | **0.635** | **3.263** | **0.122** | **2.323** |
| ***Note.*** N=290. Pooled estimates from mixed-effects linear regression analysis with 20 imputed datasets. Model 1 = ACE variable OR cortisol, adjusted for sex, age, and family SES; Model 2 = Model 1 + latent genetic risk scores. Values in bold represent statistically significant coefficients at the 5% level (p=<0.05). SE = standard error. CI = 95% confidence interval. | | | | | | | | |

| **sTable 16. Interaction effects between ACEs and average daytime cortisol levels on depressive symptoms.** | | | | | | |
| --- | --- | --- | --- | --- | --- | --- |
|  | **Estimate**  **(b)** | **SE** | **P-value** | **Lower CI** | **Upper CI** | **Effect size** |
| **ACEs cumulative score (ref: 0 ACEs)** | | |  |  |  |  |
| 1 ACE | 1.846 | 1.322 | 0.164 | -0.744 | 4.437 | 0.115 |
| 2 ACEs | 2.342 | 1.392 | 0.094 | -0.385 | 5.070 | 0.146 |
| 3 ACEs | **3.909** | **1.096** | **<0.001** | **1.762** | **6.056** | **0.244** |
| Cortisol | 0.085 | 0.189 | 0.653 | -0.285 | 0.455 | 0.005 |
| 1 ACE x Cortisol | -0.288 | 0.318 | 0.366 | -0.911 | 0.335 | -0.018 |
| 2 ACEs x Cortisol | -0.373 | 0.327 | 0.256 | -1.013 | 0.268 | -0.023 |
| 3 ACEs x Cortisol | **-0.564** | **0.292** | **0.050** | **-1.136** | **-0.000** | **-0.035** |
| **FA-derived ACEs clusters** | |  |  |  |  |  |
| Abuse | **2.268** | **1.050** | **0.033** | **0.210** | **4.327** | **0.142** |
| Cortisol | -0.161 | 0.147 | 0.278 | -0.450 | 0.128 | -0.010 |
| Abuse x Cortisol | -0.237 | 0.240 | 0.327 | -0.707 | 0.234 | -0.015 |
| Bullying | **2.078** | **1.023** | **0.044** | **0.073** | **4.084** | **0.130** |
| Cortisol | -0.152 | 0.141 | 0.282 | -0.429 | 0.124 | -0.010 |
| Bullying x Cortisol | -0.37 | 0.320 | 0.250 | -0.997 | 0.258 | -0.023 |
| Separation/Divorce | 0.499 | 1.487 | 0.738 | -2.416 | 3.413 | 0.031 |
| Cortisol | -0.211 | 0.143 | 0.142 | -0.491 | 0.069 | -0.013 |
| Separation/Divorce x Cortisol | -0.140 | 0.318 | 0.662 | -0.764 | 0.484 | -0.009 |
|  |  |  |  |  |  |  |
| Dysfunctional parenting | 1.840 | 0.957 | 0.057 | -0.037 | 3.716 | 0.115 |
| Cortisol | -0.050 | 0.179 | 0.781 | -0.402 | 0.302 | -0.003 |
| Dysfunctional parenting x Cortisol | -0.331 | 0.228 | 0.150 | -0.778 | 0.116 | -0.021 |
| **LCA-derived ACEs clusters (ref: Low ACEs)** | | |  |  |  |  |
| Dysfunctional parenting/  Emotional Abuse | 2.734 | 1.526 | 0.075 | -0.257 | 5.725 | 0.171 |
| Dysfunctional parenting | **2.149** | **1.057** | **0.044** | **0.078** | **4.220** | **0.134** |
| Separation/Divorce | -0.460 | 1.851 | 0.804 | -4.088 | 3.168 | -0.029 |
| Emotional abuse | **3.099** | **1.402** | **0.030** | **0.350** | **5.847** | **0.194** |
| Cortisol | -0.059 | 0.142 | 0.679 | -0.336 | 0.219 | -0.004 |
| Dysfunctional parenting/Emotional Abuse x Cortisol | -0.329 | 0.467 | 0.483 | -1.245 | 0.588 | -0.021 |
| Dysfunctional parenting x Cortisol | -0.390 | 0.268 | 0.148 | -0.916 | 0.136 | -0.024 |
| Separation/Divorce x Cortisol | -0.087 | 0.411 | 0.832 | -0.893 | 0.718 | -0.005 |
| Emotional abuse x Cortisol | -0.426 | 0.422 | 0.316 | -1.253 | 0.401 | -0.027 |
| ***Note.*** N=290. Pooled estimates from mixed-effects linear regression analysis with 20 imputed datasets. Model 4 – adjusted for sex, age, family SES, and latent genetic risk scores. SE = standard error. CI = 95% confidence interval. | | | | | | |

| **sTable 17. Longitudinal mediation effects of average daytime cortisol levels (age 11) in the association between ACEs and depressive symptoms (age 21).** | | | | | | | | | | | | | |
| --- | --- | --- | --- | --- | --- | --- | --- | --- | --- | --- | --- | --- | --- |
|  |  | **Mediation Effect (unexposed)** | | | | **Mediation Effect (exposed)** | | | | **Mediation Effect (average)** | | | |
| **Exposure** | **Model** | **Estimate**  **(ACME)** | **Lower CI** | **Upper CI** | **Proportion mediated effect** | **Estimate**  **(ACME)** | **Lower CI** | **Upper CI** | **Proportion mediated effect** | **Estimate**  **(ACME)** | **Lower CI** | **Upper CI** | **Proportion mediated effect** |
| ACEs cumulative score (3+ ACEs) | Model 1 | -0.019 | -0.220 | 0.193 | -0.007 | **0.614** | **0.191** | **1.198** | **0.309** | **0.298** | **0.070** | **0.604** | **0.151** |
|  | Model 2 | 0.024 | -0.085 | 0.157 | 0.015 | **0.288** | **0.032** | **0.602** | **0.218** | **0.156** | **0.018** | **0.343** | **0.116** |
| **FA-derived ACEs clusters** | |  |  |  |  |  |  |  |  |  |  |  |  |
| Abuse | Model 1 | 0.078 | -0.111 | 0.332 | 0.033 | 0.234 | -0.026 | 0.618 | 0.107 | 0.156 | -0.016 | 0.415 | 0.070 |
|  | Model 2 | 0.061 | -0.024 | 0.182 | 0.035 | 0.078 | -0.045 | 0.270 | 0.046 | 0.069 | -0.030 | 0.212 | 0.041 |
| Bullying | Model 1 | 0.141 | -0.039 | 0.381 | 0.093 | 0.313 | -0.125 | 0.930 | 0.201 | 0.227 | -0.024 | 0.551 | 0.147 |
|  | Model 2 | 0.108 | -0.017 | 0.277 | 0.117 | 0.256 | -0.015 | 0.687 | 0.282 | **0.182** | **0.011** | **0.425** | **0.199** |
| Separation/Divorce | Model 1 | -0.017 | -0.200 | 0.142 | 0.009 | -0.099 | -0.958 | 0.835 | 0.163 | -0.058 | -0.573 | 0.476 | 0.086 |
|  | Model 2 | -0.003 | -0.158 | 0.147 | 0.011 | -0.010 | -0.424 | 0.399 | 0.046 | -0.006 | -0.271 | 0.273 | 0.029 |
| Dysfunctional parenting | Model 1 | -0.073 | -0.318 | 0.076 | -0.045 | 0.197 | -0.056 | 0.540 | 0.184 | 0.062 | -0.031 | 0.225 | 0.070 |
|  | Model 2 | -0.016 | -0.129 | 0.058 | -0.009 | 0.096 | -0.103 | 0.336 | 0.132 | 0.040 | -0.038 | 0.162 | 0.061 |
| **LCA-derived ACEs clusters** | |  |  |  |  |  |  |  |  |  |  |  |  |
| Dysfunctional parenting/  Emotional Abuse | Model 1 | -0.020 | -0.275 | 0.235 | 0.009 | **0.905** | **0.049** | **1.761** | **0.355** | **0.442** | **0.039** | **0.846** | **0.182** |
|  | Model 2 | 0.022 | -0.116 | 0.160 | 0.007 | 0.308 | -0.045 | 0.661 | 0.106 | 0.165 | -0.043 | 0.372 | 0.057 |
| Dysfunctional parenting | Model 1 | 0.005 | -0.109 | 0.120 | -0.015 | -0.036 | -0.363 | 0.291 | -0.101 | -0.015 | -0.179 | 0.148 | -0.058 |
|  | Model 2 | 0.001 | -0.079 | 0.081 | 0.001 | -0.032 | -0.185 | 0.121 | 0.029 | -0.015 | -0.109 | 0.078 | 0.015 |
| Separation/Divorce | Model 1 | 0.091 | -0.182 | 0.364 | -0.004 | -0.157 | -0.909 | 0.595 | -0.024 | -0.033 | -0.404 | 0.338 | -0.014 |
|  | Model 2 | -0.023 | -0.188 | 0.143 | -0.067 | -0.375 | -0.839 | 0.089 | 0.458 | -0.199 | -0.460 | 0.062 | 0.195 |
| Emotional abuse | Model 1 | -0.019 | -0.190 | 0.151 | -0.004 | 0.006 | -0.314 | 0.326 | -0.029 | -0.007 | -0.182 | 0.168 | -0.017 |
|  | Model 2 | 0.018 | -0.076 | 0.113 | 0.016 | -0.007 | -0.230 | 0.216 | 0.004 | 0.006 | -0.122 | 0.133 | 0.010 |
| ***Note.*** N=290. Pooled estimates from mixed-effects linear regression analysis with 20 imputed datasets. Model 1 = adjusted for sex, age, and family SES; Model 2 = Model 1 + latent genetic liability for cortisol and depressive symptoms. The estimation of the mediation effect in the unexposed and exposed groups takes into account possible interactions between the exposure and mediator variables. Values in bold represent statistically significant coefficients at the 5% significance level (p<0.05). ACME = average causal mediation effect. CI = 95% confidence interval. | | | | | | | | | | | | | |

| **sTable 18. Associations of ACEs with cortisol reactivity at age 11.** | | | | | | | | |
| --- | --- | --- | --- | --- | --- | --- | --- | --- |
|  | **Model** | **Estimate** | **SE** | **P-value** | **Lower CI** | **Upper CI** | **Effect size** | **E-value** |
| **ACEs total score (ref: 0 ACEs)** | |  |  |  |  |  |  |  |
| 1 ACE | Model 1 | 0.260 | 0.285 | 0.362 | -0.299 | 0.819 | 0.029 |  |
|  | Model 2 | 0.284 | 0.279 | 0.309 | -0.263 | 0.832 | 0.029 |  |
| 2 ACEs | Model 1 | 0.078 | 0.304 | 0.799 | -0.518 | 0.673 | 0.014 |  |
|  | Model 2 | 0.119 | 0.294 | 0.686 | -0.457 | 0.694 | 0.013 |  |
| 3 ACEs | Model 1 | 0.244 | 0.253 | 0.335 | -0.251 | 0.739 | 0.022 |  |
|  | Model 2 | 0.176 | 0.250 | 0.480 | -0.313 | 0.666 | 0.019 |  |
| **FA-derived ACEs clusters** |  |  |  |  |  |  |  |  |
| Abuse | Model 1 | -0.132 | 0.238 | 0.579 | -0.598 | 0.334 | 0.021 |  |
|  | Model 2 | -0.217 | 0.231 | 0.349 | -0.670 | 0.236 | 0.021 |  |
| Bullying | Model 1 | 0.145 | 0.189 | 0.442 | -0.225 | 0.515 | -0.014 |  |
|  | Model 2 | 0.089 | 0.184 | 0.632 | -0.273 | 0.450 | -0.020 |  |
| Separation/Divorce | Model 1 | -0.033 | 0.271 | 0.905 | -0.564 | 0.499 | -0.006 |  |
|  | Model 2 | -0.030 | 0.262 | 0.910 | -0.544 | 0.485 | -0.006 |  |
| Dysfunctional parenting | Model 1 | 0.241 | 0.195 | 0.218 | -0.141 | 0.623 | 0.013 |  |
|  | Model 2 | 0.244 | 0.185 | 0.188 | -0.119 | 0.606 | 0.012 |  |
| **LCA-derived ACEs clusters (ref: Low ACEs)** | |  |  |  |  |  |  |  |
| Dysfunctional parenting/  Emotional Abuse | Model 1 | -0.579 | 0.310 | 0.063 | -1.187 | 0.029 | -0.054 |  |
|  | Model 2 | **-0.654** | **0.310** | **0.036** | **-1.261** | **-0.047** | **-0.061** | **2.492** |
| Dysfunctional parenting | Model 1 | **0.447** | **0.204** | **0.029** | **0.048** | **0.846** | **0.042** |  |
|  | Model 2 | **0.468** | **0.201** | **0.021** | **0.074** | **0.862** | **0.043** | **2.091** |
| Separation/Divorce | Model 1 | -0.197 | 0.365 | 0.590 | -0.912 | 0.519 | -0.018 |  |
|  | Model 2 | -0.168 | 0.359 | 0.640 | -0.872 | 0.536 | -0.016 |  |
| Emotional abuse | Model 1 | 0.452 | 0.276 | 0.103 | -0.089 | 0.993 | 0.042 |  |
|  | Model 2 | 0.362 | 0.277 | 0.193 | -0.182 | 0.906 | 0.034 |  |
| ***Note.*** N=290. Pooled estimates from mixed-effects linear regression analysis with 20 imputed datasets. Model 1 = adjusted for sex, age, and family SES. Model 2 = Model 1 + latent genetic liability for cortisol. Values in bold represent statistically significant coefficients at the 5% significance level (p<0.05). SE = standard error. CI = 95% confidence interval. | | | | | | | | |

| **sTable 19. Associations of cortisol reactivity with depressive symptoms at age 21.** | | | | | |
| --- | --- | --- | --- | --- | --- |
|  | **Model** | **Estimate** | **SE** | **P-value** | **95% CI** |
| Cortisol reactivity | Model 1 | -0.038 | 0.248 | 0.878 | -0.525;0.448 |
|  | Model 2 | -0.165 | 0.164 | 0.317 | -0.486;0.157 |
| **Note.** N=290. Pooled estimates from mixed-effects linear regression analysis with 20 imputed datasets. Model 1 = adjusted for sex, age, and family SES; Model 2 = Model 1 + latent genetic risk scores. | | | | | |

| **sTable 20. Longitudinal mediation effects of cortisol reactivity in the association between ACEs and depressive symptoms.** | | | | | | | |
| --- | --- | --- | --- | --- | --- | --- | --- |
|  |  | **Mediation Effect (unexposed)** | | **Mediation Effect (exposed)** | | **Mediation Effect (average)** | |
| **Exposure** | **Model** | **Estimate**  **(ACME)** | **95% CI** | **Estimate**  **(ACME)** | **95% CI** | **Estimate**  **(ACME)** | **95% CI** |
| 3+ ACEs (ref: <3 ACEs) | Model 1 | -0.083 | -0.329;0.094 | -0.006 | -0.206;0.227 | -0.044 | -0.200;0.097 |
|  | Model 2 | -0.039 | -0.151;0.045 | -0.052 | -0.19;0.033 | -0.046 | -0.156;0.027 |
| **FA-derived ACEs clusters** | |  |  |  |  |  |  |
| Abuse | Model 1 | -0.003 | -0.096;0.094 | 0.023 | -0.137;0.212 | 0.010 | -0.076;0.111 |
|  | Model 2 | 0.021 | -0.059;0.142 | 0.048 | -0.075;0.245 | 0.035 | -0.047;0.167 |
| Bullying | Model 1 | -0.006 | -0.122;0.089 | -0.059 | -0.359;0.109 | -0.033 | -0.180;0.059 |
|  | Model 2 | -0.024 | -0.132;0.047 | -0.030 | -0.198;0.088 | -0.027 | -0.140;0.052 |
| Separation/Divorce | Model 1 | 0.006 | -0.090;0.114 | 0.004 | -0.208;0.211 | 0.005 | -0.109;0.135 |
|  | Model 2 | 0.004 | -0.120;0.114 | 0.009 | -0.144;0.200 | 0.006 | -0.110;0.147 |
| Dysfunctional parenting | Model 1 | -0.059 | -0.287;0.093 | -0.010 | -0.163;0.118 | -0.035 | -0.170;0.061 |
|  | Model 2 | -0.038 | -0.183;0.062 | -0.051 | -0.184;0.027 | -0.045 | -0.158;0.018 |
| **LCA-derived ACEs clusters (ref: Low ACEs)** | | |  |  |  |  |  |
| Dysfunctional parenting/  Emotional Abuse | Model 1 | -0.013 | -0.182;0.156 | -0.075 | -0.483;0.333 | -0.044 | -0.239;0.151 |
|  | Model 2 | 0.003 | -0.118;0.124 | -0.017 | -0.278;0.244 | -0.007 | -0.140;0.127 |
| Dysfunctional parenting | Model 1 | 0.004 | -0.176;0.185 | 0.092 | -0.124;0.308 | 0.048 | -0.082;0.179 |
|  | Model 2 | -0.060 | -0.190;0.070 | 0.006 | -0.124;0.135 | -0.027 | -0.110;0.056 |
| Separation/Divorce | Model 1 | -0.041 | -0.287;0.206 | 0.160 | -0.532;0.851 | 0.059 | -0.233;0.352 |
|  | Model 2 | 0.055 | -0.121;0.230 | -0.028 | -0.393;0.336 | 0.013 | -0.178;0.204 |
| Emotional abuse | Model 1 | 0.045 | -0.101;0.190 | 0.151 | -0.132;0.433 | 0.098 | -0.055;0.250 |
|  | Model 2 | 0.053 | -0.045;0.152 | 0.032 | -0.151;0.216 | 0.043 | -0.066;0.152 |
| ***Note.*** N=290. Pooled estimates from mixed-effects linear regression analysis with 20 imputed datasets. Model 1 = adjusted for sex, age, and family SES; Model 2 = Model 1 + latent genetic liability for cortisol and depressive symptoms. The estimation of the mediation effect in the unexposed and exposed groups takes into account possible interactions between the exposure and mediator variables. Values in bold represent statistically significant coefficients at the 5% significance level (p<0.05). ACME = average causal mediation effect. CI = 95% confidence interval. | | | | | | | |

| **sTable 21. Associations of the FA-derived ACEs clusters with average daytime cortisol levels and depressive symptoms in multilevel SEM.** | | | | | |
| --- | --- | --- | --- | --- | --- |
|  |  | **Estimate (b)** | **SE** | **P-value** | **Effect size** |
| **a) Outcome: Average cortisol** | |  |  |  |  |
| Abuse | Model 1 | -1.183 | 0.783 | 0.136 | -0.255 |
|  | Model 2 | -0.534 | 0.662 | 0.422 | -0.176 |
| Bullying | Model 1 | **-0.750** | **0.330** | **0.023** | **-0.221** |
|  | Model 2 | **-0.644** | **0.297** | **0.031** | **-0.173** |
| Separation/Divorce | Model 1 | 0.575 | 1.343 | 0.672 | 0.094 |
|  | Model 2 | 0.536 | 1.040 | 0.610 | 0.079 |
| Dysfunctional parenting | Model 1 | -0.862 | 0.723 | 0.235 | -0.177 |
|  | Model 2 | -0.530 | 0.618 | 0.392 | -0.097 |
| **b) Outcome: Depressive symptoms** | |  |  |  |  |
| Abuse | Model 1 | **9.383** | **3.853** | **0.020** | **0.536** |
|  | Model 2 | **5.954** | **2.162** | **0.010** | **0.348** |
| Bullying | Model 1 | **1.823** | **0.749** | **0.020** | **0.220** |
|  | Model 2 | **1.299** | **0.622** | **0.040** | **0.124** |
| Separation/Divorce | Model 1 | 9.796 | 23.950 | 0.686 | 0.528 |
|  | Model 2 | 3.665 | 10.062 | 0.719 | 0.193 |
| Dysfunctional parenting | Model 1 | **4.369** | **2.103** | **0.043** | **0.299** |
|  | Model 2 | **2.617** | **1.294** | **0.049** | **0.172** |
| ***Note.*** N=290. Pooled estimates from multilevel structural equation models (SEM) with 20 imputed datasets. Model 1 = adjusted for sex, age, and family SES; Model 2 = Model 1 + latent genetic liability for cortisol/depressive symptoms. SE = standard error. Values in bold represent statistically significant coefficients at the 5% significance level (p<0.05). | | | | | |

| **sTable 22. Associations of the individual ACEs items with average daytime cortisol levels and depressive symptoms.** | | | | | | |
| --- | --- | --- | --- | --- | --- | --- |
|  | **Model** | **Estimate** | **SE** | **P-value** | **Lower CI** | **Upper CI** |
| **a) Outcome: Average Cortisol** | |  |  |  |  |  |
| Negative Parental Practices | Model 1 | -0.550 | 0.282 | 0.053 | -1.103 | 0.004 |
|  | Model 2 | -0.426 | 0.246 | 0.084 | -0.908 | 0.055 |
| Negative Parental Feelings | Model 1 | -0.169 | 0.263 | 0.520 | -0.685 | 0.346 |
|  | Model 2 | -0.130 | 0.220 | 0.556 | -0.561 | 0.302 |
| Maternal Depression | Model 1 | 0.111 | 0.282 | 0.693 | -0.441 | 0.663 |
|  | Model 2 | 0.081 | 0.236 | 0.731 | -0.381 | 0.544 |
| Divorce | Model 1 | -0.035 | 0.456 | 0.940 | -0.927 | 0.858 |
|  | Model 2 | 0.006 | 0.398 | 0.987 | -0.774 | 0.787 |
| Separation | Model 1 | 0.421 | 0.620 | 0.497 | -0.794 | 1.636 |
|  | Model 2 | 0.422 | 0.523 | 0.420 | -0.602 | 1.446 |
| Bullying | Model 1 | **-0.611** | **0.227** | **0.008** | **-1.056** | **-0.166** |
|  | Model 2 | **-0.510** | **0.204** | **0.013** | **-0.910** | **-0.111** |
| Emotional abuse | Model 1 | -0.337 | 0.321 | 0.295 | -0.967 | 0.292 |
|  | Model 2 | -0.054 | 0.284 | 0.849 | -0.610 | 0.502 |
| Physical abuse | Model 1 | -0.518 | 0.540 | 0.338 | -1.576 | 0.540 |
|  | Model 2 | -0.291 | 0.463 | 0.530 | -1.199 | 0.617 |
| **b) Outcome: Depressive symptoms** | |  |  |  |  |  |
| Negative Parental Practices | Model 1 | 1.332 | 0.775 | 0.089 | -0.186 | 2.850 |
|  | Model 2 | 0.882 | 0.567 | 0.122 | -0.229 | 1.993 |
| Negative Parental Feelings | Model 1 | 0.733 | 0.903 | 0.420 | -1.037 | 2.503 |
|  | Model 2 | 0.538 | 0.547 | 0.328 | -0.535 | 1.611 |
| Maternal Depression | Model 1 | 1.586 | 0.825 | 0.058 | -0.032 | 3.203 |
|  | Model 2 | 0.538 | 0.492 | 0.277 | -0.426 | 1.502 |
| Divorce | Model 1 | -0.686 | 1.609 | 0.671 | -3.840 | 2.469 |
|  | Model 2 | -0.488 | 0.927 | 0.600 | -2.304 | 1.328 |
| Separation | Model 1 | 0.930 | 1.507 | 0.539 | -2.023 | 3.883 |
|  | Model 2 | 0.390 | 0.951 | 0.683 | -1.474 | 2.254 |
| Bullying | Model 1 | **1.860** | **0.854** | **0.032** | **0.186** | **3.534** |
|  | Model 2 | **1.047** | **0.473** | **0.030** | **0.119** | **1.974** |
| Emotional abuse | Model 1 | 1.670 | 0.938 | 0.079 | -0.168 | 3.507 |
|  | Model 2 | **1.329** | **0.607** | **0.031** | **0.139** | **2.518** |
| Physical abuse | Model 1 | **4.630** | **1.902** | **0.017** | **0.902** | **8.357** |
|  | Model 2 | **3.029** | **1.232** | **0.016** | **0.615** | **5.443** |
| ***Note.*** N=290. Pooled estimates from mixed-effects linear regression analysis with 20 imputed datasets. Model 1 = adjusted for sex, age, and family SES. Model 2 = Model 1 + latent genetic liability for cortisol. Values in bold represent statistically significant coefficients at the 5% significance level (p<0.05). SE = standard error. CI = 95% confidence interval. | | | | | | |

| **sTable 23. Longitudinal mediation effects of average daytime cortisol levels (age 11) in the association between ACEs and depressive symptoms (age 21) - Adjusted for depressive symptoms at age 11.** | | | | | | | |
| --- | --- | --- | --- | --- | --- | --- | --- |
|  |  | **Mediation Effect (unexposed)** | | **Mediation Effect (exposed)** | | **Mediation Effect (average)** | |
| **Exposure** | **Model** | **Estimate** | **95% CI** | **Estimate** | **95% CI** | **Estimate** | **95% CI** |
| ACEs cumulative score (3+ ACEs) | Model 1 | -0.014 | -0.232;0.203 | **0.580** | **0.064;1.096** | **0.283** | **0.061;0.586** |
|  | Model 2 | 0.030 | -0.097;0.158 | **0.298** | **0.022;0.575** | **0.164** | **0.020;0.371** |
| **FA-derived ACEs clusters** |  |  |  |  |  |  |  |
| Abuse | Model 1 | 0.088 | -0.092;0.268 | 0.216 | -0.153;0.584 | 0.152 | -0.061;0.365 |
|  | Model 2 | 0.061 | -0.040;0.162 | 0.079 | -0.109;0.267 | 0.070 | -0.060;0.200 |
| Bullying | Model 1 | 0.132 | -0.094;0.359 | 0.299 | -0.208;0.806 | 0.216 | -0.005;0.528 |
|  | Model 2 | 0.117 | -0.033;0.266 | 0.275 | -0.071;0.620 | **0.196** | **0.016;0.470** |
| Separation/Divorce | Model 1 | -0.008 | -0.245;0.229 | -0.038 | -0.594;0.517 | -0.023 | -0.364;0.318 |
|  | Model 2 | -0.004 | -0.157;0.149 | -0.008 | -0.292;0.276 | -0.006 | -0.199;0.187 |
| Dysfunctional parenting | Model 1 | -0.055 | -0.204;0.093 | 0.175 | -0.115;0.464 | 0.060 | -0.097;0.217 |
|  | Model 2 | -0.012 | -0.105;0.082 | 0.090 | -0.083;0.264 | 0.039 | -0.070;0.149 |
| **LCA-derived ACEs clusters (ref: Low ACEs)** |  |  |  |  |  |  |  |
| Dysfunctional parenting/  Emotional Abuse | Model 1 | 0.004 | -0.294;0.303 | 0.664 | -0.287;1.615 | 0.334 | -0.107;0.776 |
|  | Model 2 | 0.041 | -0.106;0.187 | 0.319 | -0.002;0.640 | 0.180 | -0.004;0.364 |
| Dysfunctional parenting | Model 1 | -0.019 | -0.157;0.118 | -0.119 | -0.359;0.121 | -0.069 | -0.199;0.061 |
|  | Model 2 | -0.044 | -0.134;0.045 | -0.120 | -0.297;0.057 | -0.082 | -0.200;0.035 |
| Separation/Divorce | Model 1 | 0.006 | -0.248;0.260 | -0.016 | -0.772;0.740 | -0.005 | -0.349;0.339 |
|  | Model 2 | -0.054 | -0.220;0.112 | -0.286 | -0.753;0.182 | -0.170 | -0.416;0.076 |
| Emotional abuse | Model 1 | -0.011 | -0.210;0.188 | -0.104 | -0.490;0.281 | -0.058 | -0.263;0.147 |
|  | Model 2 | 0.016 | -0.070;0.102 | 0.019 | -0.171;0.209 | 0.017 | -0.087;0.122 |
| ***Note.*** N=290. Pooled estimates from mixed-effects linear regression analysis with 20 imputed datasets. Model 1 = adjusted for sex, age, maternal education, and depressive symptoms at age 11; Model 2 = Model 1 + latent genetic liability for cortisol and depressive symptoms. The estimation of the mediation effect in the unexposed and exposed groups takes into account possible interactions between the exposure and mediator variables. Values in bold represent statistically significant coefficients at the 5% significance level (p<0.05). | | | | | | | |

| **sTable 24. Associations of ACEs with average daytime cortisol levels and cortisol reactivity (age 11) - Complete data analysis.** | | | | | | | | | |
| --- | --- | --- | --- | --- | --- | --- | --- | --- | --- |
|  |  | **Average daytime cortisol** | | | | **Cortisol reactivity** | | | |
|  | **Model** | **Estimate**  **(b)** | **SE** | **Lower CI** | **Upper CI** | **Estimate**  **(b)** | **SE** | **Lower CI** | **Upper CI** |
| **ACEs cumulative score (ref: 0 ACEs)** | | | | | | | | | |
| 1 ACE | Model 1 | -0.236 | 0.341 | -0.904 | 0.433 | 0.124 | 0.285 | -0.435 | 0.683 |
|  | Model 2 | -0.163 | 0.300 | -0.752 | 0.425 | 0.097 | 0.285 | -0.462 | 0.656 |
| 2 ACEs | Model 1 | -0.435 | 0.380 | -1.181 | 0.310 | 0.249 | 0.314 | -0.367 | 0.864 |
|  | Model 2 | -0.072 | 0.336 | -0.731 | 0.588 | 0.188 | 0.317 | -0.434 | 0.810 |
| 3 ACEs | Model 1 | -0.818 | 0.506 | -1.809 | 0.174 | -0.296 | 0.389 | -1.058 | 0.466 |
|  | Model 2 | -0.546 | 0.434 | -1.397 | 0.305 | -0.339 | 0.389 | -1.101 | 0.423 |
| **FA-derived ACEs dimensions** | |  |  |  |  |  |  |  |  |
| Abuse | Model 1 | -0.535 | 0.305 | -1.132 | 0.062 | -0.072 | 0.267 | -0.596 | 0.452 |
|  | Model 2 | -0.155 | 0.272 | -0.689 | 0.379 | -0.203 | 0.276 | -0.744 | 0.337 |
| Bullying | Model 1 | -0.406 | 0.327 | -1.046 | 0.234 | 0.177 | 0.265 | -0.343 | 0.697 |
|  | Model 2 | -0.340 | 0.286 | -0.900 | 0.220 | 0.174 | 0.264 | -0.344 | 0.691 |
| Separation/Divorce | Model 1 | 0.235 | 0.589 | -0.919 | 1.390 | 0.360 | 0.428 | -0.480 | 1.199 |
|  | Model 2 | 0.332 | 0.493 | -0.633 | 1.298 | 0.355 | 0.428 | -0.484 | 1.194 |
| Dysfunctional parenting | Model 1 | 0.194 | 0.387 | -0.564 | 0.952 | 0.268 | 0.282 | -0.285 | 0.822 |
|  | Model 2 | 0.222 | 0.316 | -0.398 | 0.842 | 0.263 | 0.280 | -0.286 | 0.812 |
| **LCA-derived ACEs clusters (ref: Low ACEs)** | | |  |  |  |  |  |  |  |
| Dysfunctional parenting/  Emotional Abuse | Model 1 | -0.419 | 0.400 | -1.203 | 0.365 | -0.559 | 0.313 | -1.172 | 0.053 |
|  | Model 2 | -0.156 | 0.355 | -0.852 | 0.539 | **-0.633** | **0.312** | **-1.245** | **-0.020** |
| Dysfunctional parenting | Model 1 | -0.129 | 0.277 | -0.671 | 0.414 | **0.526** | **0.208** | **0.118** | **0.933** |
|  | Model 2 | -0.097 | 0.242 | -0.571 | 0.377 | **0.536** | **0.206** | **0.133** | **0.939** |
| Separation/Divorce | Model 1 | 0.601 | 0.503 | -0.385 | 1.587 | -0.196 | 0.365 | -0.912 | 0.519 |
|  | Model 2 | 0.455 | 0.435 | -0.397 | 1.307 | -0.171 | 0.360 | -0.876 | 0.535 |
| Emotional abuse | Model 1 | **-0.937** | **0.351** | **-1.626** | **-0.249** | 0.424 | 0.282 | -0.129 | 0.977 |
|  | Model 2 | -0.528 | 0.317 | -1.149 | 0.093 | 0.330 | 0.284 | -0.227 | 0.887 |
| ***Note.*** N=163. Estimates from mixed-effects linear regression analysis. Model 1 = adjusted for sex, age, and family SES; Model 2 = Model 1 + latent genetic liability for cortisol. | | | | | | | | | |

| **sTable 25. Associations of ACEs, average daytime cortisol levels, and cortisol reactivity (age 11) with depressive symptoms (age 21) - Complete data analysis.** | | | | | |
| --- | --- | --- | --- | --- | --- |
|  | **Model** | **Estimate**  **(b)** | **SE** | **Lower CI** | **Upper CI** |
| **ACEs cumulative score (ref:0 ACEs)** | | |  |  |  |
| 1 ACE | Model 1 | 0.011 | 0.818 | -1.593 | 1.615 |
|  | Model 2 | 0.574 | 0.524 | -0.453 | 1.601 |
| 2 ACEs | Model 1 | 1.336 | 0.912 | -0.451 | 3.124 |
|  | Model 2 | **1.277** | **0.583** | **0.135** | **2.419** |
| 3 ACEs | Model 1 | 2.280 | 1.209 | -0.090 | 4.651 |
|  | Model 2 | **1.982** | **0.771** | **0.471** | **3.493** |
| **FA-derived ACEs clusters** | |  |  |  |  |
| Abuse | Model 1 | **1.547** | **0.764** | **0.049** | **3.045** |
|  | Model 2 | **1.866** | **0.462** | **0.961** | **2.770** |
| Bullying | Model 1 | **1.720** | **0.740** | **0.268** | **3.171** |
|  | Model 2 | **1.150** | **0.495** | **0.180** | **2.120** |
| Separation/Divorce | Model 1 | -0.964 | 1.385 | -3.677 | 1.750 |
|  | Model 2 | -0.635 | 0.891 | -2.381 | 1.111 |
| Dysfunctional parenting | Model 1 | 0.814 | 0.886 | -0.922 | 2.551 |
|  | Model 2 | 0.571 | 0.585 | -0.576 | 1.718 |
| **LCA-derived ACEs clusters (ref: Low ACEs)** | | |  |  |  |
| Dysfunctional parenting/  Emotional Abuse | Model 1 | 2.189 | 1.156 | -0.076 | 4.454 |
|  | Model 2 | **1.840** | **0.712** | **0.445** | **3.236** |
| Dysfunctional parenting | Model 1 | 0.697 | 0.904 | -1.075 | 2.469 |
|  | Model 2 | 0.685 | 0.554 | -0.402 | 1.772 |
| Separation/Divorce | Model 1 | -2.759 | 1.860 | -6.403 | 0.886 |
|  | Model 2 | -1.265 | 2.152 | -5.482 | 2.953 |
| Emotional abuse | Model 1 | 1.256 | 1.038 | -0.777 | 3.290 |
|  | Model 2 | **2.292** | **0.645** | **1.028** | **3.556** |
| **Average daytime cortisol** | Model 1 | -0.356 | 0.193 | -0.734 | 0.023 |
|  | Model 2 | -0.271 | 0.143 | -0.550 | 0.009 |
| **Cortisol reactivity** | Model 1 | -0.230 | 0.228 | -0.530 | 0.362 |
|  | Model 2 | -0.084 | 0.145 | -0.515 | 0.055 |
| ***Note.*** N=163. Estimates from mixed-effects linear regression analysis. Model 1 = ACE measure OR cortisol, adjusted for sex, age, and family SES; Model 2 = Model 1 + latent genetic risk scores. | | | | | |
